# MyoD is essential in rhabdomyosarcoma by promoting survival through differentiation and CYLD

**DOI:** 10.1016/j.isci.2025.113149

**Published:** 2025-07-18

**Authors:** Alexander R. Oles, Peter Y. Yu, Abasi-ama Udeme, Sudarshana Sharma, Priya Londhe, Benjamin R. Pryce, Erin E. Talbert, Eric M. Hill, Carlos J. Miranda, Brian K. Kaspar, Michael A. Arnold, Jack Hyland, Cheryl A. London, Peter J. Houghton, David J. Wang, Ryan D. Roberts, Denis C. Guttridge

**Affiliations:** 1 Department of Pediatrics, Darby Children’s Research Institute, Medical University of South Carolina, Charleston, SC 29425, USA; 2 Department of Cancer Biology and Genetics, The Ohio State University Medical Center, Columbus, OH 43210, USA; 3 Hollings Cancer Center, Medical University of South Carolina, Charleston, SC 29425, USA; 4 Department of Health and Human Physiology, and the Holden Comprehensive Cancer Center, University of Iowa, Iowa City, IA 52242, USA; 5 Department of Pediatrics, Center for Gene Therapy, Nationwide Children’s Hospital, Columbus, OH 43205, USA; 6 Department of Pathology and Laboratory Medicine, Children’s Hospital Colorado, Aurora, CO 80045, USA; 7 Department of Pathology and Laboratory Medicine, University of Colorado, Anschutz Medical Campus, Aurora, CO 80045, USA; 8 Cummings School of Veterinary Medicine, Tufts University, Medford, MA 02155, USA; 9 Greehey Children’s Cancer Research Institute, University of Texas, San Antonio, TX 23220, USA; 10 Department of Pediatrics, Center for Childhood Cancer, Nationwide Children’s Hospital, Columbus, OH 43205, USA

**Keywords:** Cell biology, Cancer

## Abstract

Rhabdomyosarcoma (RMS) is the most common soft tissue cancer among children, characterized by a skeletal muscle lineage that is impaired from undergoing terminal differentiation. NF-κB is constitutively active in cancer cells and plays a critical role in cell survival. Although NF-κB is also activated in RMS, surprisingly, we find that these tumors are far less dependent on NF-κB for their survival. Instead, RMS cells survive, paradoxically, by being partially differentiated under the control of the myogenic transcription factor MyoD. Loss of MyoD, or cellular reprogramming, dedifferentiates RMS tumor cells and sensitizes their death under stress. MyoD enhances RMS survival by regulating DNA methyltransferases, which in turn suppresses the tumor suppressor and pro-apoptotic gene CYLD. From these findings, we propose that MyoD acts as an oncogene in RMS by enhancing survival through pro-differentiation and anti-cell death activities.

## Highlights

• RMS cell survival is not dependent on NF-κB
• MyoD promotes RMS survival by maintaining cells in a partially differentiated state
• MyoD regulates DNA methyltransferases to suppress CYLD-mediated cell death
• MyoD exhibits oncogenic activities in RMS fusion positive and negative subtypes

## Graphical abstract

**Figure:**
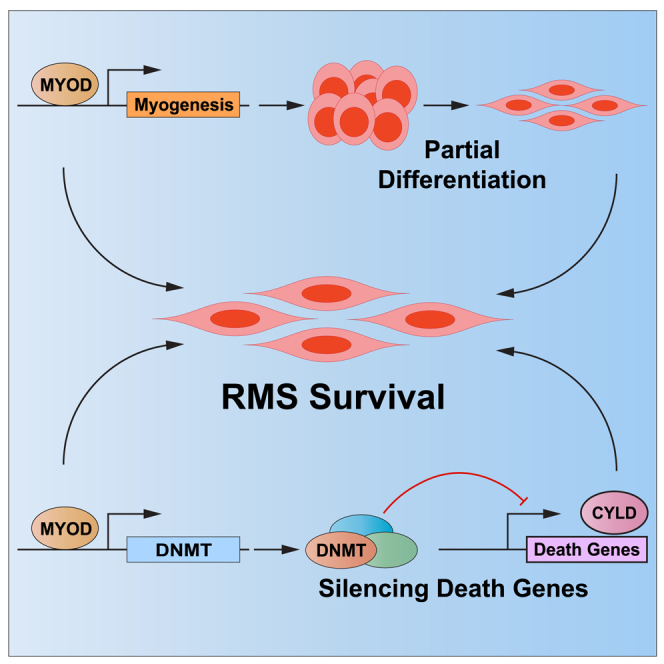

## Introduction

Rhabdomyosarcoma (RMS) is a soft-tissue sarcoma diagnosed in approximately 350 children and teenagers in the United States each year.^1^^,^^2^^,^^3^ Multimodal therapy is curative in most cases, but for patients with high-risk RMS, the 5-year survival rate is less than 20%, a level that has not changed in the last 40 years^4^^,^^5^^,^^6^ Recent World Health Organization guidelines recognize four histological subtypes of RMS, but the majority of cases in the pediatric population are either embryonal (ERMS) or alveolar (ARMS) RMS.^2^^,^^7^ These two subtypes often have distinct clinical characteristics, but more recently, genetics have contributed in distinguishing their diagnosis and risk-stratification for treatment protocols.^8^^,^^9^ While ERMS tumors account for a majority of cases, ARMS tumors are much more clinically aggressive, implying a difference in underlying biology.^3^

ERMS is genetically diverse, with a greater mutational burden and aneuploidy rate than the ARMS subtype.^10^ ERMS mutations typically involve loss of heterozygosity at chromosome 11p15 or mutations in the *RAS*, *FGFR4*, *TP53*, *NF1*, *PIK3CA*, *BCOR*, or *FBXW7* gene that correlate with worse outcomes.^10^^,^^11^ Conversely, nearly 80% of ARMS tumors contain an oncogenic fusion gene consisting of the DNA binding domain of myogenic homeobox transcription factors, *PAX3* or *PAX7*, and the transactivation domain of FOXO1, resulting in a *PAX3::FOXO1* or *PAX7::FOXO1* fusion gene.^12^ The presence of the fusion gene itself has been implicated as a marker of poor survival in patients.^13^^,^^14^ As such, the denotation of fusion positive (FP) or fusion negative (FN) RMS is now a standard reference because of its clinical meaningfulness.^4^^,^^10^ Additionally, a recent international effort linked the presence of *TP53* or *MYOD1* mutations to poor outcomes in intermediate-risk group patients.^4^^,^^10^

While the two subtypes vary in driver mutations, phenotypically, all RMS subtypes are thought to share origins with skeletal muscle or its precursor tissue.^15^ In order to diagnose a sarcoma as RMS, there must be evidence of embryonic skeletal myogenesis, as indicated by expression of myogenic regulatory factors (MRFs) and other skeletal myogenic markers such as desmin or myosin.^16^^,^^17^ MRFs are a family of four basic-helix-loop-helix E-box binding transcription factors which include MyoD (MYOD1), Myf5, myogenin, and MRF4 (Myf6).^18^^,^^19^ These factors function in concert to control the stepwise process of skeletal muscle determination and differentiation.^20^^,^^21^ Unlike normal skeletal muscle, terminal differentiation is impaired in RMS cells due to the perturbation of the MRF transcriptional network, preventing growth arrest and myofiber formation.^22^^,^^23^ Although recent comprehensive genomic analyses support the hypothesis that RMS tumor cells are partially differentiated along the skeletal muscle lineage,^24^^,^^25^ a longstanding paradigm in the field has been that overcoming the differentiation block may provide a therapeutic strategy for this lethal pediatric cancer that would be applicable to all RMS subtypes.^22^^,^^23^

Our own laboratory conformed to this paradigm and previously reported that the classical signaling pathway of NF-κB is constitutively active in RMS and contributes to this pathology by inhibiting differentiation, analogous to how classical NF-κB impairs skeletal myogenesis.^26^^,^^27^ However, because NF-κB functions generally in oncogenesis as a regulator of survival,^28^^,^^29^^,^^30^^,^^31^^,^^32^^,^^33^ we asked whether NF-κB survival activity regulates RMS pathogenesis. Surprisingly, we discovered that unlike seemingly every other solid or hematopoietic tumor cell, RMS cells do not require NF-κB to survive. Instead, RMS tumors are protected from cytotoxic or genotoxic stress by their partially differentiated state induced and maintained by MyoD, but not other MRFs. We further find that MyoD mediates RMS cell survival by directly regulating DNA methyltransferases (DNMT), linked to the repression of the tumor suppressor and pro-apoptotic gene, cylindromatosis (CYLD). These results challenge the longstanding paradigm for how MyoD functions in RMS pathogenesis.

## Results

### RMS tumor cells resist stress-induced cell death independent of NF-κB

**Figure 1:**
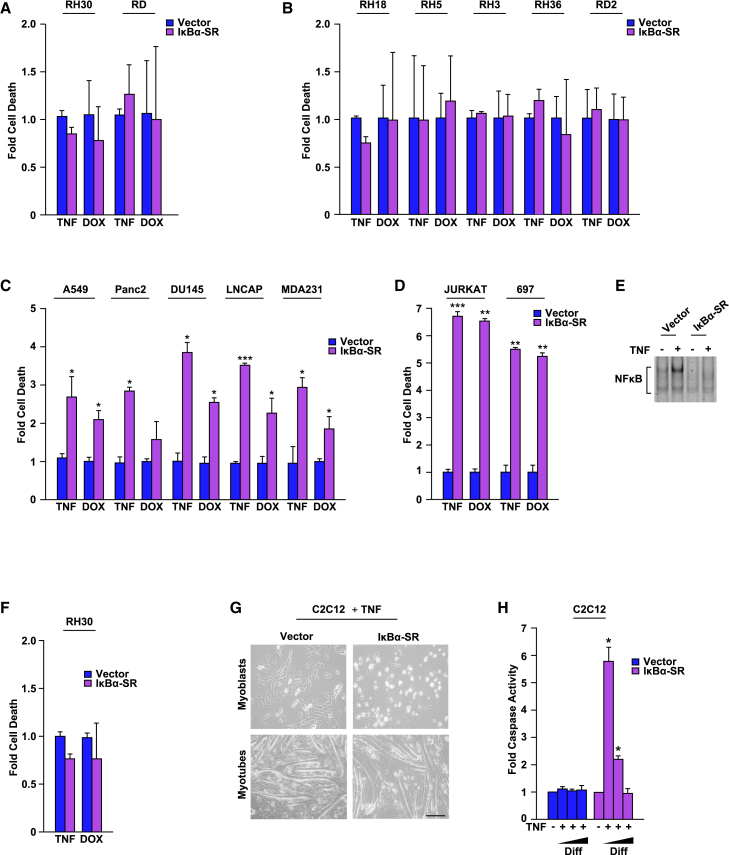
RMS cells resist stress-induced cell death independent of NF-κB (A and B) FP and FN RMS cancer cells expressing a vector control or the IκBα super repressor (IκBα-SR) inhibitor of NF-κB were treated with TNF (5 ng/mL) or DOX (1 μM) for 24 h and cells were subsequently analyzed for cell death by Annexin V using flow cytometry, *n* = 3. (C and D) Similar analysis as described in (A and B) was performed on epithelial (C) and hematopoietic (D) tumor cells, *n* = 3. (E and F) The inhibition of NF-κB DNA binding activity in RH30 tumor cells stably expressing the IκBα-SR was analyzed by electrophoretic mobility shift assays and cell death was analyzed following treatment with TNF, *n* = 3. (G) Phase-contrast images of C2C12 vector and IκBα-SR stably expressing murine myoblasts and myotubes treated with TNF for 12 h; scale bar represents 100 μm. (H) C2C12 vector and IκBα-SR myoblasts induced to differentiate (Diff) for 0, 48 or 96 h and then treated with TNF for 24 h (+), were subsequently analyzed for cell death by caspase 3 cleavage activity and compared using one-way ANOVA with Dunnett’s multiple comparisons, *n* = 3. Data with error bars are depicted as mean ± SEM, ∗*p* < 0.05; ∗∗*p* < 0.01; ∗∗∗*p* < 0.001. See also Figure S1.

We previously showed that NF-κB is constitutively active in RMS, and functions by impairing differentiation through epigenetic silencing of the pro-myogenic microRNA, miR-29.^27^^,^^34^ In normal muscle, this miR contributes to skeletal myogenesis and prevents RMS by targeting the anti-myogenic transcription factor YY1 and silencing the pro-tumorigenic activity of the mRNA binding protein HuR, respectively. In addition to inhibiting differentiation, we asked whether NF-κB could also function in RMS as a survival factor. To make this determination, we virally transduced FP (alveolar; RH30) and FN (embryonal; RD) RMS cells with a transdominant inhibitor of NF-κB, referred to as the IκBα-Super Repressor (IκBα-SR; Figure S1A).^35^ Vector control and IκBα-SR expressing cells were subsequently stressed with the addition of the cytokine, tumor necrosis factor (TNF; 5 ng/mL), or the chemotherapeutic compound, doxorubicin (DOX; 1 μM), both of which accentuate apoptosis in cells lacking NF-κB.^33^ To our surprise, IκBα-SR expressing RH30 and RD cells were resistant to TNF and DOX mediated killing, as compared to vector control cells, and irrespective of the vehicle (Figure 1A). This was not cell line specific, as similar results were confirmed with other RMS FP (RH3, RH5, RH18, and RH36), and FN (RD2) tumor cells (Figure 1B). As a control, a similar approach was taken with a panel of epithelial (A549 – lung, Panc2 – pancreas, DU145 – prostate, LNCAP – prostate, MDA231 – breast) and hematopoietic (Jurkat – T cell, 697 – B cell) tumor lines (Figures S1B and S1C). As anticipated, and supported by a widely accepted literature,^31^^,^^36^ epithelial and lymphoid tumor cells expressing IκBα-SR were sensitive to cytokine and genotoxic stress-induced apoptosis (Figures 1C and 1D). To ensure the inactivation of NF-κB in RMS cells, we clonally selected an RH30 cell line stably expressing the IκBα-SR transgene and tested for NF-κB activity. Despite nearly complete absence of NF-κB DNA binding (Figure 1E), RMS cells continued to resist stress mediated killing by TNF or DOX (Figure 1F). Furthermore, doubling the concentrations of TNF on RH30 IκBα-SR cells did not significantly increase cell death compared to similarly treated MDA231 IκBα-SR breast cancer cells (Figure S1D). This resistance to stress was also not specific to DOX, since RH30 IκBα-SR cells were resistant to other chemotherapeutic compounds, etoposide and camptothecin (Figure S1E). To account for any nonspecific effects of the IκBα-SR transgene, NF-κB was also inhibited using an siRNA targeting the p65 subunit of the transcription factor in A549 lung cancer cells and RH30 cells (Figures S1F and S1G). Knockdown of p65 in A549 cells significantly enhanced TNF-induced cell death, while having little to no effect on survival in RH30 cells (Figure S1H). Together, these data reveal that unlike epithelial and hematopoietic tumors, RMS possesses a unique ability to overcome a susceptibility to stress-induced cell death in the absence of NF-κB.

Since RMS is linked to a myogenic lineage, we asked whether a similar survival mechanism existed in skeletal muscle. C2C12 myoblasts stably expressing IκBα-SR were visibly more sensitive to TNF-induced cell death than vector control (Figure 1G). However, once differentiated into myotubes, IκBα-SR expressing C2C12 cells appeared resistant to cell death. To determine if this difference was due to differentiation of myoblasts, we induced vector and IκBα-SR expressing C2C12 myoblasts to differentiate for various times and subsequently added TNF for 24 h. Whereas TNF again sensitized IκBα-SR expressing myoblasts, cell death was significantly reduced in differentiating IκBα-SR cells (Figure 1H). This resistance to cell death in differentiated muscle was not due to a post-mitotic condition, since IκBα-SR expressing C2C12 myoblasts forced to exit the cell cycle with mitomycin C treatment remained sensitive to TNF (Figure S1I). Cell-cycle arrest was confirmed with BrdU staining and cell growth curves (Figures S1J and S1K). Collectively, these results suggest that differentiated myocytes resist cell death when lacking NF-κB signaling.

### RMS survival is mediated by the selective activity of MyoD

**Figure 2:**
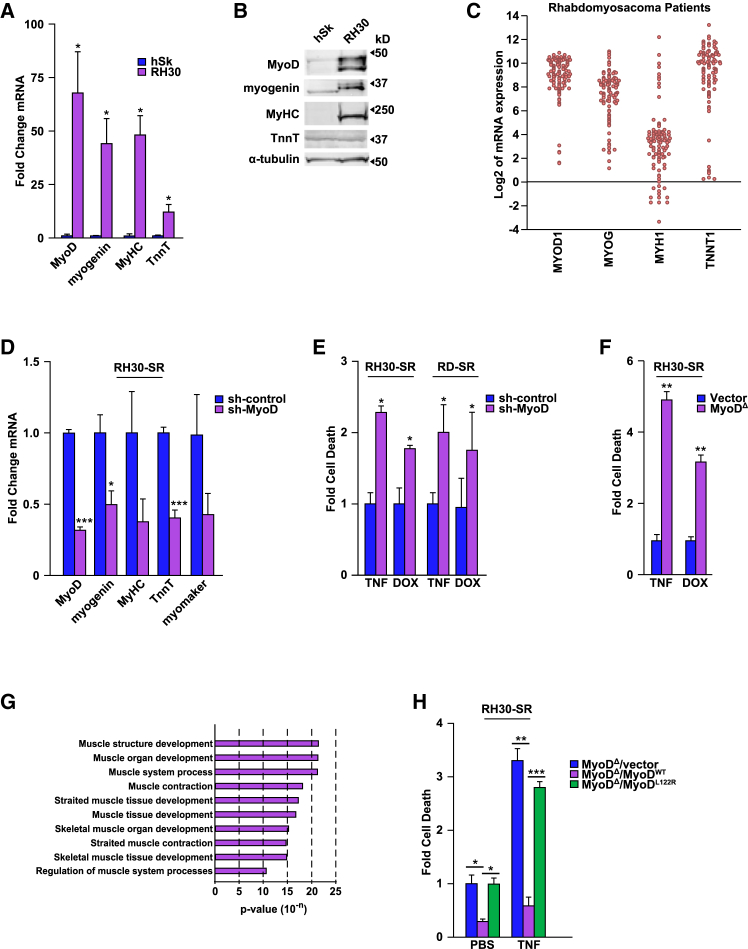
RMS cells are resistant to cell death due to a differentiation phenotype (A and B) Myogenic gene expression was measured by qPCR and western blot analyses in human skeletal muscle myoblasts (hSk) and RH30 cells, *n* = 3. (C) Abundance of myogenic genes, *MYOD1, MYOG, MYH1(MYHC),* and *TNNT1* from 101 RMS patients retrieved from an R2 Genomics Analysis and Visualization Platform. (D) qPCR analysis comparing expression of MyoD and its respective target genes in myogenic differentiation in RH30-SR cells containing a scrambled-control sh-RNA or sh-RNA targeting MyoD, *n* = 3. (E) Cells from (D) were treated with TNF or DOX, and cell death was subsequently measured by flow cytometry, *n* = 3. (F) RH30-SR cells expressing scrambled small guide RNAs (Vector) or a knockdown of MyoD generated by CRISPR/Cas9 deletion (MyoD^Δ^), were treated with TNF or DOX, and subsequently measured for cell death, *n* = 3. (G) GO enrichment analysis for genes with decreased expression in RH30-SR MyoD^Δ^ compared to RH30-SR Vector cells. (H) RH30-SR MyoD^Δ^ cells were reconstituted with control (Vector), the wildtype (WT) form of MyoD, or a mutant form of MyoD (MyoD^L122R^). Cells were then exposed to either PBS or TNF for 24 h and cell death was measured and normalized to the untreated RH30-SR MyoD^Δ^ cells. Treatment groups were analyzed with one-way ANOVAs with Tukey’s multiple comparisons *n* = 3. Data with error bars are depicted as mean ± SEM, ∗*p* < 0.05; ∗∗*p* < 0.01; ∗∗∗*p* < 0.001. See also Figure S2.

Although RMS tumors are considered incapable of completing terminal differentiation, given our results obtained in differentiated muscle cells, we questioned whether the unique ability of RMS cells to resist cell death when lacking NF-κB related to their muscle lineage.^27^^,^^34^ To test this, we probed for expression of myogenic differentiation markers in RH30 cells. The mRNA and protein levels of MRFs, MyoD and myogenin, and contractile protein, myosin heavy chain (MyHC), were markedly greater in RH30 cells compared to human muscle myoblasts, with similar findings with troponin T (TnnT) mRNA (Figure 2A). Although expression of MyoD and myogenin in RMS is well documented,^16^^,^^17^ the drastic difference in expression of MyHC in these cells was surprising. To probe whether clonal variance among RH30 cells accounted for elevated levels of myogenic genes, we performed single cell qRT-PCR analyses. Results from violin plots showed that levels of MyoD, myogenin, MyHC, and TnnT were uniformly expressed in RH30 cells (Figure S2A and Table S1). A further investigation of an RMS patient dataset,^37^^,^^38^ analyzed by the R2 Genomics Analysis and Visualization Platform, showed expression of terminally differentiated myogenic genes, including MyoD and myogenin (Figure 2C). Together, these results reveal that RMS tumors exhibit characteristics of terminal differentiation.

Next, we tested whether, similar to C2C12 myotubes, this differentiation phenotype of RMS cells could serve as a survival mechanism in the absence of NF-κB. Since MyoD is a master regulator of muscle differentiation,^18^^,^^19^^,^^39^ we depleted this factor with a targeted sh-RNA (sh-MyoD) in FP and FN IκBα-SR expressing cells (RH30-SR and RD-SR; Figure S2B). We confirmed that knocking down MyoD reduced expression of differentiation genes, myogenin, MyHC, TnnT, and myomaker (Figure 2D). Significantly, knocking down MyoD in RH30-SR and RD-SR cells also reversed resistance to cytokine and genotoxic mediated killing (Figure 2E). Deletion of MyoD using CRISPR/Cas9 gene editing (MyoD^Δ^; Figure S2C) produced an even greater stress-induced cell death response (expressed as both relative and absolute cell death, respectively; Figures 2F, S2D, and S2E). This again correlated with reduced expression of differentiation genes (Figure S2F), which we further verified by performing a transcriptomic analysis comparing MyoD^Δ^ to control cells. Gene Ontology (GO) analysis revealed that downregulated genes, due to loss of MyoD, associated with pathways of muscle contraction and differentiation (Figure 2G). These results support the conclusion that decreasing differentiation of RMS cells leads to stress-induced cell death. To further test the specificity of MyoD in regulating cell survival, we reconstituted MyoD^Δ^ cells with a wildtype copy of MyoD (MyoD^WT^; Figure S2G). Results showed that MyoD^WT^ rescued death in RH30-SR MyoD^Δ^ cells (Figure 2H), but a similar rescue was not achieved with the MyoD mutant, *MYOD1*^L122R^ which impairs myoblast differentiation.^40^

**Figure 3:**
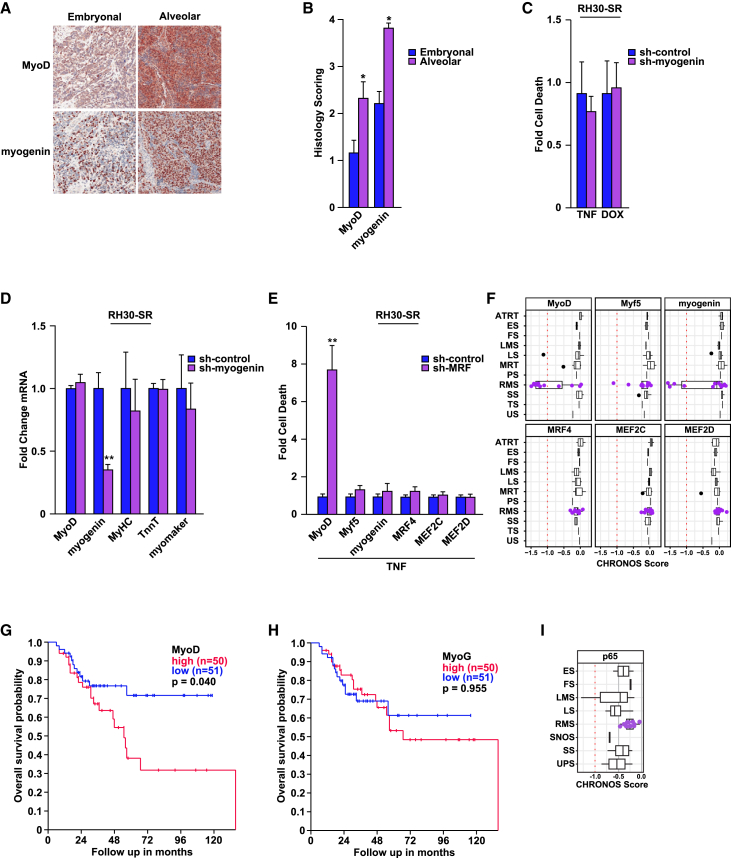
RMS survival is selectively regulated by MyoD (A) Representative images of immunohistochemistry staining of MyoD and myogenin in patient embryonal and alveolar tumors. (B) Histologic images in (A) were scored for MyoD and myogenin immunohistochemistry staining positivity, *n* = 5. (C) RH30-SR cells expressing a scrambled-control sh-RNA or sh-RNA targeting myogenin were treated with TNF and DOX for 24 h and subsequently measured for cell death, *n* = 3. (D) RNA was prepared from cells in (C) and qPCR analysis was performed probing for myogenin and its target genes, *n* = 3. (E) RH30-SR cells expressing a scrambled-control sh-RNA or sh-RNA against either MyoD, MYF5, myogenin, MRF4, MEF2C, and MEF2D (sh-MRFs) were treated with TNF for 24 h and cell death was subsequently measured, *n* = 3. (F) CHRONOS Score for MRFs in sarcoma cell lines tested in DepMap. RMS cell lines are highlighted in purple, with a score of < −1 indicating a gene that is essential; ATRT, atypical teratoid rhabdoid tumors; ES, Ewing sarcoma; FS, follicular sarcoma; LMS, leiomyosarcoma; LS, liposarcoma; MRT, malignant rhabdoid tumor; PS, pleomorphic sarcoma; RMS, rhabdomyosarcoma; SS, synovial sarcoma; TS, thyroid sarcoma; US, undifferentiated sarcoma. (G and H) Kaplan-Meier curve, log rank test, showing the correlation of both (G) MyoD and (H) myogenin expression stratified by median patient expression to overall survival of rhabdomyosarcoma patients from an R2 Genomics Analysis and Visualization Platform. (I) CHRONOS Score for p65 in sarcoma cell lines tested in the Cancer Dependency Map. Data with error bars are depicted as mean ± SEM, ∗*p* < 0.05; ∗∗*p* < 0.01. See also Figure S3.

Myogenin belongs to the same family of skeletal muscle-specific transcription factors as MyoD.^18^^,^^19^ Although both MRFs are expressed in RMS alveolar/FP and embryonal/FN subtypes,^17^ immunohistochemical staining of patient samples showed that expression of MyoD and myogenin was significantly higher in the more aggressive and treatment-resistant alveolar/FP subtype (Figures 3A, 3B, and S3A). A similar increase in MyoD and myogenin was demonstrated in alveolar/FP and embryonal/FN Orthotopic-Patient Derived Xenografts (O-PDXs) (Figures S3B and S3C). We hypothesized from these results that myogenin should phenocopy the survival function of MyoD in RMS cells exposed to stress. However, when myogenin was depleted with a targeted sh-RNA (sh-myogenin) in RH30-SR cells, results showed that these cells remained resistant to stress (FigureS 3C and S3D). In addition, although myogenin shares similar functions to MyoD in regulating myogenesis, myogenin knockdown in RH30-SR cells had no significant effect on MyHC, TnnT, or myomaker gene expression (Figure 3D). This was confirmed by performing single cell qRT-PCR analysis where we observed that unlike MyoD whose expression in RH30 cells correlates highly with expression of MyHC and TnnT, this correlation is profoundly reduced for myogenin (Figure S3E). Therefore, in contrast to MyoD, myogenin does not function as a survival factor in RMS tumor cells due to its lack of regulation of differentiation. Since myogenesis is also regulated by MRFs, Myf5 and MRF4, as well as cooperative myocyte enhancing factors (MEF2),^20^^,^^21^ we asked if any of these factors could contribute to RMS survival. Using a similar sh-RNA targeting strategy, results showed that only the loss of MyoD, but not Myf5, MRF4, MEF2C, or MEF2D, sensitized RMS cells to stress-induced cell death (Figures 3E and S3F). Our data were further supported by DepMap results that showed in a panel of RMS cell lines that MyoD had the strongest dependence on cell survival compared to other MRFs (Figure 3F).^41^^,^^42^ Using the same RMS patient dataset as described in Figure 2, we also observed that high expression of MyoD, but not myogenin or other MRFs or MEF2 factors, correlated with significantly worse patient survival outcomes (Figures 3G, 3H, and S3G).^38^ Notably, when RMS cell lines were ranked based on their dependency on MyoD, RH30 scored at −1.29 (Figure S3H), an indication that these tumor cells are dependent on MyoD (<−1.0). Yet, when we deleted MyoD from RH30 cells, we observed only minimal effects on survival and proliferation, compared to vector cells (Figures S3I and S3J). In contrast, similar deletion of MyoD from RD and RH4 cells, whose Chronos scores on DepMap are −1.55 and −1.58, respectively, caused significantly more death, and to the best of our abilities we were unsuccessful in maintaining these cells in culture, indicative of their high dependence on MyoD for survival activity (Figure S3K). Furthermore, RMS cells showed no dependency on the p65 subunit of NF-κB (Figure 3I), which supports our earlier conclusion that survival of these tumors is independent of NF-κB.

### RMS survival is mediated through a partial differentiation phenotype

**Figure 4:**
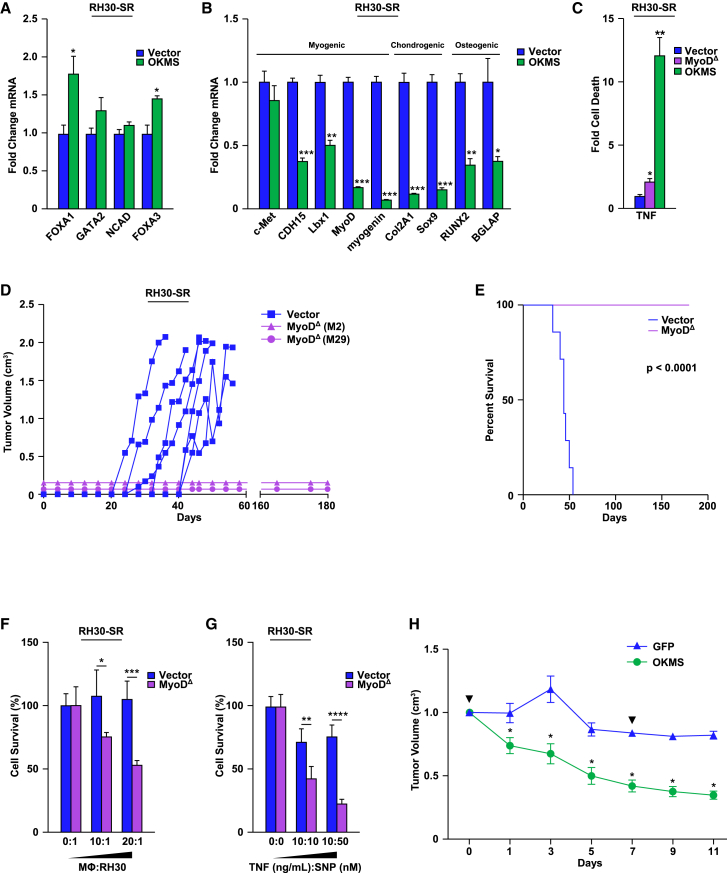
RMS tumors utilize MyoD to resist stress *in vivo* (A) RH30-SR cells were infected with Vector-control or Yamanaka reprogramming factors (OKMS) and expression of stem cell factors was analyzed by qPCR, *n* = 3. (B) RH30-SR cells were infected as detailed in (A) and analyzed by qPCR for mesenchymal genes, *n* = 3. (C) Cells in (A) were treated with TNF for 12 h and cell death was measured as compared to RH30-SR MyoD^Δ^ cells using one-way ANOVA with Dunnett’s multiple comparisons, *n* = 3. (D) RH30-SR MyoD^Δ^ (clones M29 and M2) and RH30-SR Vector control cells were injected in SCID mice and tumor volumes were measured over time, *n* = 5. (E) A Kaplan-Meier curve, log rank test, was generated to predict the overall survival of mice with RH30-SR Vector and RH30-SR MyoD^Δ^ tumors, *n* = 5. (F) RH30-SR Vector and RH30-SR MyoD^Δ^ cells were co-cultured with activated macrophages (0:1, 10:1, and 20:1) and cell survival was subsequently scored by trypan blue exclusion and compared to baseline cell death, analyzed with a two-way ANOVA with Sidak’s multiple comparisons with an interaction of *p* = 0.0133, *n* = 3. (G) RH30-SR Vector and RH30-SR MyoD^Δ^ cells were treated with 0 or 10 ng/mL of TNF and 0, 10, or 50 nM of sodium nitroprusside (SNP) and cell survival was subsequently scored and analyzed as in (F), with an interaction of *p* = 0.0003, *n* = 3. (H) RH30-SR cells were administered to SCID mice and tumors that formed were subsequently injected with lentiviruses expressing OKMS factors or GFP control. Mice were then treated with 2 doses of vincristine (1.0 mg/kg; days 0 and 7; arrowheads) and tumor volumes were continually measured, *n* = 5. Data with error bars are depicted as mean ± SEM, ∗*p* < 0.05; ∗∗*p* < 0.01; ∗∗∗*p* < 0.001; ∗∗∗∗*p* < 0.0001. See also Figure S4.

If differentiation provides a survival advantage for RMS tumors, we speculated that forcing RMS cells to exit the myogenic lineage into a more primitive, pluripotent state should render these cells even more sensitive to dying in the presence of stress. To test this, we infected RH30-SR cells with lentiviruses expressing the Yamanaka factors (Oct3/4, Klf4, c-Myc, Sox2; referred to as OKMS) (Figure S4A) to induce cellular reprogramming.^43^ Compared to control cells, expression of OKMS factors caused the induction of some stem cells markers, *FoxA1 and FoxA3* (Figure 4A), as well as the reduced expression of mesenchymal lineage genes (myogenesis, chondrogenesis, and osteogenesis) (Figure 4B). This showed that RH30-SR cells were successfully reprogrammed. Importantly, treatment of reprogrammed RH30-SR cells with TNF caused a dramatic increase in cell death, a level 6-fold higher than what was achieved with TNF treated RH30-SR MyoD^Δ^ cells (Figure 4C). This supported the notion that RMS cells depend on their differentiation phenotype as a survival mechanism against stress.

To examine the *in vivo* relevance of our findings, we injected RH30-SR MyoD^Δ^ cells into SCID mice, expecting that a stronger biological stress under *in vivo* conditions would consequently affect tumor development due to the absence of both NF-κB and MyoD. While SCID mice injected with RH30-SR vector cells produced tumors that reached endpoint at a median of 42 days (Figures 4D and 4E), mice injected with two separate RH30-SR MyoD^Δ^ clonally selected cell lines failed to develop tumors even after 180 days of observation. This inability to form tumors was not due to a proliferation defect since growth in culture was comparable to vector cells (Figure S4B). Furthermore, soft agar colony formation assays revealed no difference between RH30-SR and RH30-SR MyoD^Δ^ cells in anchorage independent growth (Figure S4C), suggesting that deletion of MyoD in these cells did not affect their transformation potential.

The ability of tumor cells to overcome immune surveillance is essential for tumor development.^44^ Because SCID mice retain an innate immune system that includes inflammatory macrophages secreting TNF,^45^ we speculated that the failure of RH30-SR MyoD^Δ^ tumors to form in mice was related to the sensitivity to the biological stress caused by infiltrating immune cells. To test this, we injected RH30-SR MyoD^Δ^ cells into the peritoneum of SCID mice. Results showed that equal amounts of macrophages were recruited in response to injection of RH30-SR MyoD^Δ^ compared to of RH30-SR vector control cells (Figure S4D). Next, we co-cultured RH30-SR MyoD^Δ^ cells with increasing ratios of primary, activated inflammatory macrophages (0:1–20:1). Results showed that RH30-SR MyoD^Δ^ cells were significantly more sensitive to macrophage-mediated killing compared to vector control RH30-SR cells (Figure 4F). Findings also showed that this sensitivity could be recapitulated with increasing ratios of secreted macrophage factors, TNF and nitric oxide (SNP) (Figure 4G), and further, that such killing was attenuated when RH30-SR MyoD^Δ^ cells were co-cultured with *TNF*^−/−^ macrophages (Figure S4E). Thus, it is likely that *in vivo*, developing RMS tumors use MyoD to resist stress from the immune surveillance activity of pro-inflammatory macrophages.

Since we observed that RH30-SR vector cells could establish tumors, we next asked whether such tumors could be sensitized to stress upon their dedifferentiation, as shown *in vitro*. Therefore, we repeated subcutaneous injections of RH30-SR cells in SCID mice, and once tumors were palpable, intratumorally injected retroviruses expressing the OKMS reprogramming factors, using GFP virus as a control. Results showed that intra-tumoral expression of OKMS factors reprogrammed tumors by reducing levels of differentiation factors, *MyoD*, *myogenin*, and *TnnT1* (Figure S4F). To test the translational potential of these findings, mice were treated with standard of care chemotherapy, vincristine, one week post retrovirus injections. Compared to GFP-injected tumors, reprogrammed RH30-SR tumors responded to vincristine administration, and tumor regression continued throughout the course of treatment (Figure 4H). Collectively, these *in vivo* results are consistent with *in vitro* findings and strongly suggest that RMS tumors resist cell death through MyoD regulating a partially differentiated phenotype.

### MyoD directly regulates DNA methyltransferases to inhibit death genes in RMS

To gain further insight on the mechanism by which MyoD regulates RMS survival, we mined publicly available ChIP-seq datasets from the ENCODE Consortium and examined genome-wide coverage of MyoD in RMS tumor cells.^46^ When genes enriched for MyoD peaks were queried for pathway enrichment analysis, surprisingly, networks of stemness-related pathways (PSCs; pluripotent stem cells) were more prominently displayed than pathways related to myogenesis, muscle movement, and muscle contraction (Figure S5A). Examination of these stemness networks revealed genes with MyoD enrichment peaks corresponding to stemness genes *CDH2*, *FOXA1*, *FOXA3, Hes1,* and *Zeb1*. From this *in silico* evidence we considered that MyoD might function as a negative regulator of stemness genes to maintain RMS cells in a partially differentiated state. However, after repeated attempts, we were unable to confirm that stemness genes we had identified could be uniformly repressed by MyoD (Figure S5B).

**Figure 5:**
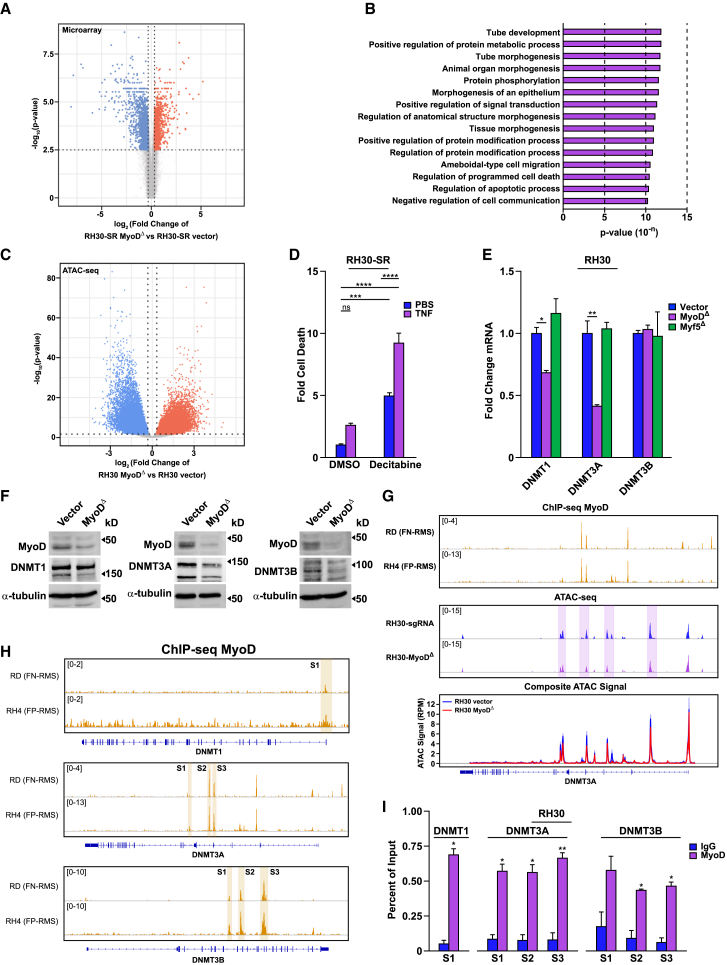
MyoD suppresses death genes through DNA methyltransferases (A) Volcano plot from transcriptomic analysis representing downregulated (blue, *n* = 1358) and upregulated genes (red, *n* = 1116) from RH30-SR MyoD^Δ^ cells compared to RH30-SR cells (fold change >25%, FDR <0.05). (B) GO analysis of upregulated genes in RH30-SR MyoD^Δ^ cells compared to RH30-SR cells. (C) Volcano plot from ATAC-seq analysis representing differentially accessible chromatin—closed (blue) and open (red)—in RH30-SR MyoD^Δ^ cells compared to RH30-SR cells (fold change >25%, FDR <0.05). (D) Cell death was measured following the treatment of RH30-SR cells with decitabine (1 μM) and TNF (5 ng/mL) compared to RH30-SR control cells treated with DMSO and PBS. Significance was determined through two-way ANOVA analysis, with Sidak’s multiple comparisons, *n* = 3. (E) Expression levels of DNMT1, DNMT3A, and DNMT3B in RH30 MyoD^Δ^ and RH30 Myf5^Δ^ compared to RH30-Vector control cells analyzed by one-way ANOVA with Dunnett’s multiple comparisons, *n* = 3. (F) Western blots of MyoD, DNMT1, DNMT3A, and DNMT3B in RH30-SR Vector or MyoD^Δ^ cells, using α−tubulin as a loading control. Arrowheads point to respective DNMTs. (G) MyoD ChIP-seq data identifying enrichment peaks on the DNMT3A gene from FN (RD) and FP (RH4) RMS cells, graphed above representative ATAC-seq data revealing loss of chromatin accessibility signal in highlighted regions of interest in RH30 MyoD^Δ^ (purple) compared to RH30 Vector cells (blue) with accompanying composite plot of ATAC-seq signals of both conditions, *n* = 3. Signal is represented as reads per million mapped reads (RPM). (H) Visualization of MyoD ChIP-seq data with enrichment peaks identified as S1-S3 on DNMT1, DNMT3A and DNMT3B genes in FN (RD) and FP (RH4) RMS cells. (I) MyoD ChIP analysis performed, as percent of input, on regions S1–S3 in DNMT1, DNMT3A and DNMT3B genes, *n* = 2. Data with error bars are depicted as mean ± SEM, ∗*p* < 0.05; ∗∗*p* < 0.01; ∗∗∗*p* < 0.001; ∗∗∗∗*p* < 0.0001. See also Figure S5.

We then took an alternative approach and compared transcriptomes between RH30-SR and RH30-SR MyoD^Δ^ cells to identify RMS genes regulated by MyoD, but independent of NF-κB survival activity. Of the 2,474 differentially expressed genes (DEGs, FC > 25%, FDR <0.05), 1,358 were downregulated and 1,116 were upregulated, which represented genes that were activated and repressed by MyoD, respectively (Figure 5A). We then combined transcriptomics with MyoD ChIP-seq datasets obtained from RH4 and RD cell lines,^47^^,^^48^ and observed that of the 1,116 upregulated genes, 82% in RH4 and 60% in RD cells lacked MyoD enrichment peaks (Figure S5C). This was revealing given that MyoD is an established transcriptional activator that binds to E box consensus sites in promoters and enhancers of myogenic genes.^39^ Such results indicated that MyoD can function in RMS as a transcriptional repressor independent of DNA binding. To gain greater insight into the identity of these 1,116 repressed genes, we used GO pathway analysis. Interestingly, regulators of cell death and apoptotic pathways were notably enriched (Figure 5B), implying that MyoD might enhance RMS cell survival through the suppression of cell death-related genes. Upon additional analysis, the 160 death genes identified belonged to the regulation of programmed cell death ontology (GO:0043067). Further annotation using the UCSC genome browser revealed that 123/160, or 77%, of these death genes contained CpG islands, compared to 747/1116, or 67%, of all upregulated genes (Figure S5D).^49^^,^^50^ Since we previously described that MyoD is capable of altering the chromatin status during skeletal muscle differentiation,^51^ we assessed if chromatin remodeling occurs upon deletion of MyoD by performing an ATAC-seq analysis between RH30-SR and RH30-SR MyoD^Δ^ cells. Results revealed that there was an approximately equal distribution of opened (8,624) and closed (8,483) chromatin regions upon loss of MyoD (Figure 5C). However, within the 160 death genes identified, 97/160, or 60%, exhibited areas of chromatin in a more open state (Figure S5E), and of those 97 death genes, 84/97, or 87%, contained CpG islands (Figure S5E).

The methylation of CpG islands is controlled by DNMTs as an epigenetic mechanism to repress gene expression.^52^ Based on our multiomic analysis, we hypothesized that MyoD regulates RMS survival by inhibiting pro-death genes through DNMTs. Therefore, we treated RH30-SR cells with the DNMT inhibitor, decitabine (5-aza-2-deoxycytidine). Results showed that RMS cells were susceptible to decitabine, and cell death was significantly enhanced following the addition of TNF (Figure 5D). Since DNMT3A was identified as a downregulated gene in our RH30-SR MyoD^Δ^ transcriptomic dataset (Figure S5F), we examined whether MyoD was a direct transcriptional activator of DNMTs. In RH30 MyoD^Δ^ cells, DNMT1 and DNMT3A mRNA were significantly reduced, as compared to control cells (Figure 5E). In contrast, no changes were seen with levels of DNMT3B. Similar findings with respect to DNMT1 mRNA were observed in RH5 MyoD^Δ^ cells (Figure S5G). In addition, regulation of DNMTs appeared specific to MyoD since similar regulation was not seen in RH30 Myf5^Δ^ cells (Figure 5E). Furthermore, regulation of DNMTs by MyoD was also observed at the protein level (Figure 5F). Moreover, analysis of RMS MyoD ChIP-seq datasets revealed MyoD enrichment peaks throughout the DNMT3A gene in FP (RH4) and FN (RD) RMS cells (Figure 5G). This was supported by our own ATAC-Seq assays, which showed that regions of chromatin mapping to the DNMT3A gene were less accessible in RH30 MyoD^Δ^ compared to vector cells, with several regions aligning with ChIP-seq MyoD peaks (Figure 5G, with composite, and Figure S5H). Further analysis of RMS ChIP-seq datasets identified MyoD binding sites on the DNMT1 promoter and on several regions of the DNMT3B gene (Figure 5H). ChIP assays confirmed the binding of MyoD on multiple sites of all three DNMT genes (Figure 5I). Together, these results suggest that MyoD regulates DNMT expression to mediate the suppression of death genes in RMS tumor cells.

### Identification of CYLD as a MyoD regulated gene mediating RMS survival

**Figure 6:**
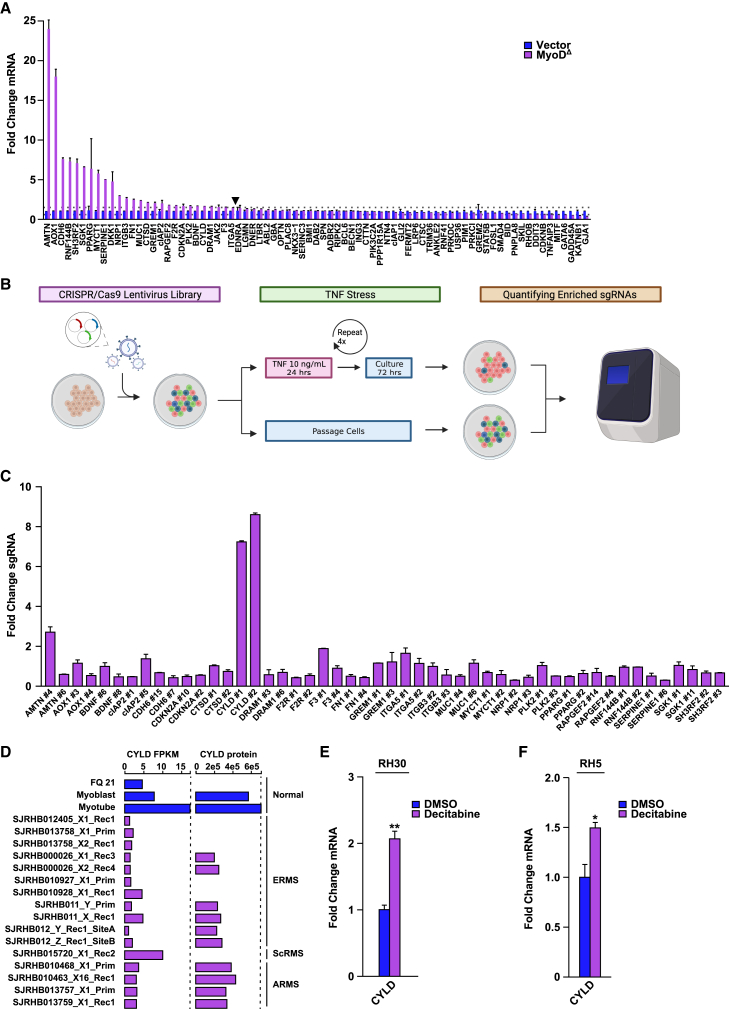
Identification of CYLD as a target of MyoD mediating RMS survival (A) qPCR analysis of 77 death related genes in RH30-SR MyoD^Δ^ cells compared to Vector control, represented as technical duplicates. Arrowhead denotes the top 25 expressed genes, *n* = 1. (B) Illustration depicting a targeted CRISPR/Cas9 screen to identify genes involved in mediating survival of RH30-SR MyoD^Δ^ RMS cells, created with BioRender.com. (C) PCR results from enrichment of targeted sgRNA guides in RH30-SR MyoD^Δ^ cells infected with 0.5 virus particles per cell following four cycles of TNF treatment for 24 h compared to control untreated cells, represented as technical duplicates, *n* = 1. (D) RNA and protein levels of CYLD from myoblasts and myotubes compared to FN/ERMS and FP/ARMS tumors, from the St. Jude Pediatric Tumor Gene Expression Dataset. Dotted line denotes the maximum expression of CYLD from cultured muscle cells. (E and F) CYLD expression in RH30 (E) and RH5 (F) cells following treatment of decitabine compared to DMSO control, *n* = 3. Data with error bars are depicted as mean ± SEM, ∗*p* < 0.05; ∗∗*p* < 0.01. See also Figure S6.

To identify which death genes are repressed by MyoD to mediate RMS survival, we combined an *in-silico* analysis with a CRISPR Cas9 knockdown screen. From 160 death genes that were originally found to be upregulated in RH30-SR MyoD^Δ^ cells, genes were filtered by the following criteria: (1) those that were upregulated >1.6-fold; (2) those that did not contain MyoD peaks; and (3) those genes that contained CpG islands. We also included genes that were upregulated in RH30-SR MyoD^Δ^ cells belonging to other related pathways (autophagy, senescence, etc.) that fit these same criteria. Ultimately, 77 death related genes were identified and probed by qRT-PCR to identify genes repressed by MyoD (Figure 6A). That analysis narrowed the number of genes to 25, whose expression was upregulated >1.5-fold in RH30-SR MyoD^Δ^ cells compared to vector control (Figures 6A and S6A). We then designed two pairs of sgRNAs for each of these 25 target genes and cloned these into lentiviruses to generate a mini sgRNA library. This CRISPR library was used to infect RH30-SR MyoD^Δ^ cells (at 0.5 virus particles per cell) stably expressing Cas9. Following limited selection, we performed 4 cycles of stress with TNF treatment for 24 h (Figure 6B). Using this dosing scheme, we speculated that RH30 cells expressing sgRNA to target pro-death genes repressed by MyoD would be enriched in MyoD knockdown cells, as these RMS cells lacking MyoD would become resistant from TNF-induced cell death. After TNF treatment, qPCR was utilized to analyze sgRNA encoding sequences from genomic DNA extracted from both TNF treated and control cells. Results revealed significant enrichment for the CYLD gene (Figure 6C). A separate screen was repeated, with the exception that ∼16 virus particles per cell were utilized to account for the possible interaction of multiple gene interactions. Following TNF treatment and qPCR amplification, surviving cells were again enriched for CYLD (Figure S6B). A third screen was performed to test the validity of our findings by increasing the duration of TNF (10 ng/mL) to 48 h. Compared to scrambled sgRNA, infections with targeted sgRNAs led to significant survival, and once again amplifications of those sequences corresponded to CYLD (Figure S6C). Combined, these results identify CYLD as a putative repressed target of MyoD mediating RMS survival.

To address the relevance of CYLD in RMS, we investigated its expression levels from the St. Jude Pediatric Tumor Gene Expression Dataset. Results showed that across multiple patient FP/ARMS and FN/ERMS tumors, CYLD was repressed relative to myoblasts and myotubes (Figure 6D). However, by ATAC-seq we observed that depleting MyoD from RH30 cells did not have a general effect on chromatin accessibility in the 5′ region of the CYLD gene (Figure S6D). Given that MyoD regulates DMNTs and CYLD was predicted to contain a CpG island, we then analyzed methylation of the CYLD gene using the Cancer Cell Line Encyclopedia (CCLE) dataset, generated from the Illumina Infinium HumanMethylation450 (450K) BeadChip array platform, which targets 96% of CpG islands in the human genome.^53^ The CYLD promoter contains a CpG island measuring approximately 1Kb. However, methylation in this island was low or undetectable in multiple RMS cell lines (Figure S6E). In contrast, the 450K array platform identified three distinct methylation sites downstream of the CYLD promoter occupied in multiple RMS cells. Since the last two of these sites are located >18Kb outside the CYLD gene, we focused on the most proximal site to the promoter. We employed bisulfite PCR analysis on 6 loci between introns 6 and 9 from RH30 vector and RH30 MyoD^Δ^ cells. From these loci, 5 were successfully amplified and cloned. Locus 3 and 5, which both exhibited a relatively high degree of methylation, showed reduced methylation in RH30 MyoD^Δ^ cells compared to RH30 vector cells (Figure S6E). The remaining loci with low to moderate levels of methylation showed no observable dependence on MyoD. Together, these data suggested that the CYLD gene is methylated under the control of MyoD. Consistent with our bisulfite sequencing data, RH30 and RH5 RMS cells treated with decitabine showed elevated expression of CYLD (Figures 6E and 6F).

### MyoD targets CYLD to mediate survival in RMS by modulating necroptosis

**Figure 7:**
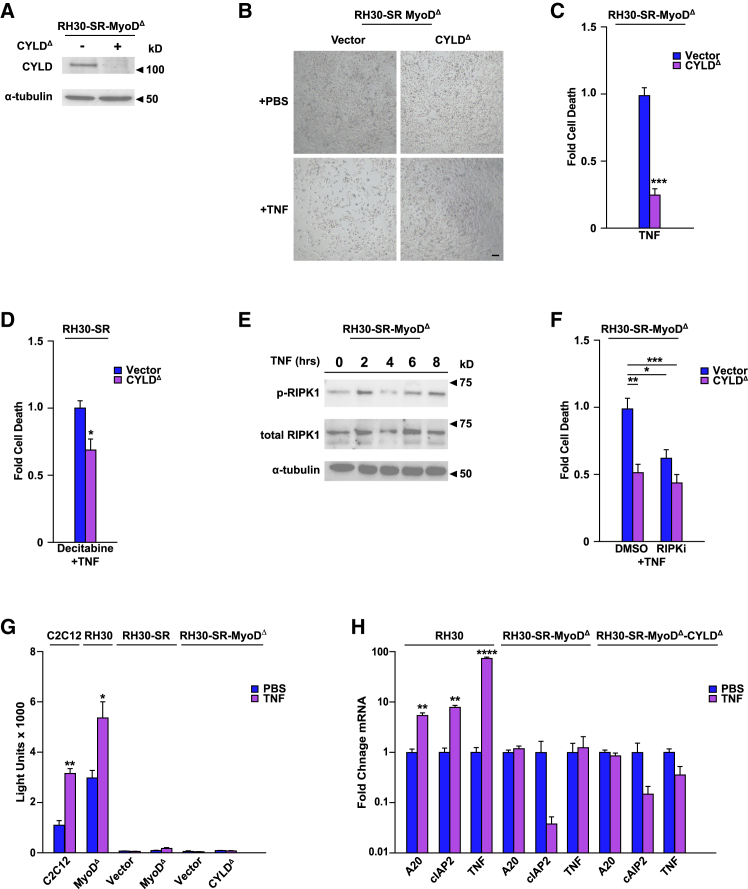
CYLD mediates TNF-induced cell death in RMS cells lacking MyoD (A) CYLD was depleted in RH30-SR MyoD^Δ^ cells using CRISPR/Cas9 gene editing, and a western blot was performed to probe for CYLD expression. (B) RH30-SR MyoD^Δ^ Vector and RH30-SR MyoD^Δ^ CYLD^Δ^ cells were treated with PBS or TNF for 24 h and cell viability was observed by phase contrast microscopy, scale bar denotes 100 μm. (C) Similar conditions as in (B), but cells were analyzed for cell death by Annexin V and flow cytometry, *n* = 3. (D) RH30-SR CYLD^Δ^ cells were treated with decitabine and TNF, *n* = 2, and cell death was compared to Vector control cells, *n* = 3. (E) RH30-SR MyoD^Δ^ cells were treated with TNF at indicated times and probed by Western for pRIPK1 and total RIPK1, with α−tubulin used as a loading control. (F) RH30-SR MyoD^Δ^ Vector and RH30-SR MyoD^Δ^ CYLD^Δ^ cells were treated with100 nM of RIPK1 inhibitor GSK963 (RIPKi) or DMSO in the presence of TNF for 24 h and analyzed for cell death. Significance was determined through two-way ANOVA analysis, with Sidak’s multiple comparisons. The interaction between groups was not significant, *n* = 3. (G) NF-κB transcriptional activity was measured in RH30-SR Vector, RH30-SR MyoD^Δ^, RH30-SR MyoD^Δ^ Vector, and RH30-SR MyoD^Δ^ CYLD^Δ^ cells using an NF-κB-Luciferase reporter plasmid in the presence of TNF compared to PBS as a control. C2C12 and RH30 MyoD^Δ^ were used as control cell lines, *n* = 3. (H) qPCR assays were performed probing for NF-κB regulated genes A20, cIAP2, and TNF in RH30, RH30-SR MyoD, and RH30-SR MyoD CYLD^Δ^ cells treated with TNF or PBS used as a control, *n* = 3. Data with error bars are depicted as mean ± SEM, ∗*p* < 0.05; ∗∗*p* < 0.01; ∗∗∗*p* < 0.001; ∗∗∗∗*p* < 0.0001. See also Figure S7.

In addressing the functional relevance of CYLD in RMS survival, western blot confirmed that RH30-SR MyoD^Δ^ cells that survived TNF stress and enriched for CYLD sgRNAs were depleted in CYLD protein (Figure 7A). RH30-SR MyoD^Δ^ cells were then infected with both pairs of CYLD sgRNA and treated with TNF to validate viability. Compared to scrambled sgRNAs (Vector) infected cells where TNF induced significant cell death, CYLD knockdown (CYLD^Δ^) exhibited a significant rescue on cell viability (Figures 7B and 7C). Notably, cell death induced by treating RH30-SR cells with TNF and decitabine was also rescued by CYLD^Δ^ (Figure 7D).

Because CYLD function has mainly been studied in the context of TNF signaling,^54^ we asked whether CYLD could mediate RMS survival in response to other stressors. RH30-SR MyoD^Δ^ and RH30-SR MyoD^Δ^ CYLD^Δ^ cells were treated with DOX or other chemotherapeutics, vincristine, actinomycin D, etoposide, or camptothecin, and tested for viability. In contrast to the viability rescued by CYLD^Δ^ in TNF treated in RH30-SR MyoD^Δ^ cells, CYLD^Δ^ was unable to rescue the viability of RH30-SR MyoD^Δ^ cells following chemotherapeutics treatments (Figure S7A). Together, these findings suggest that MyoD suppression of CYLD leads to RMS survival in response to TNF stress-induced killing.

CYLD is a tumor suppressor mutated in familial cylindromatosis, a genetic condition that predisposes patients to neoplasms of the skin, and more recently, mutations in CYLD have been described in other cancers.^55^^,^^56^ CYLD functions as a deubiquitinase (DUB) and promotes cell death by two major mechanisms; one through the cleavage of polyubiquitin linkages on RIPK1, which activates the kinase and upregulates RIP3 to promote necroptosis, and second by deubiquitinating several upstream regulators of the NF-κB signaling pathway, which inhibits cell survival through the activation of caspase mediated apoptosis.^54^ To further explore the functional relevance of CYLD in RMS, we examined downstream targets of CYLD when cell death is induced in response to TNF in RH30-SR MyoD^Δ^ cells. Results showed that RH30-SR MyoD^Δ^ cells were capable of phosphorylating RIPK1 when treated with TNF (Figure 7E). However, cells were partially rescued from death following treatment with a RIPK1 inhibitor (Figure 7F), suggesting that MyoD regulates CYLD in RMS to limit necroptosis. When we then examined effects on NF-κB signaling in RH30-SR MyoD^Δ^ cells treated with TNF, no significant changes were observed compared to RH30 cells, either by assaying NF-κB reporter activity or NF-κB target genes (Figures 7G and 7H). Such data demonstrated that the IκBα-SR functionally maintains NF-κB inhibition, regardless of CYLD function. Together, these findings suggest that in response to cytokine stress, RMS survival is dependent on the suppression of CYLD, mediated by DNMTs under the control of MyoD.

## Discussion

In this study, we initially probed into the relevance of the survival activity of NF-κB in RMS, but in the end discovered new mechanisms by which these cancer cells resist conditions of stress that might be meaningful to RMS pathogenesis. Our findings highlight the following conclusions: (1) In contrast to most epithelial and hematopoietic tumor cells that require NF-κB activity for survival, RMS cells, which are of mesenchymal lineage, are less dependent on NF-κB for survival; (2) in lieu of NF-κB, RMS tumor cells depend on their partially differentiated phenotype as a resistance mechanism against stress; (3) survival of RMS tumor cells through their differentiation phenotype is mediated by MyoD; (4) besides maintaining RMS tumors in a partially differentiated state, MyoD also functions in these tumors as a repressor of death related genes; (5) repression of these death genes is associated with CpG methylation and MyoD regulation of DNMTs; and (6) the tumor suppressor, CYLD, represents one type of death gene whose repression in RMS is mediated by MyoD, and required to promote RMS survival in response to cytokine stress.

Studies from the late 1990s deduced that NF-κB functions as a survival factor through direct transcriptional regulation of anti-apoptotic genes.^36^ Soon after, it was surmised that tumor cells treated with chemotherapeutic agents that activated NF-κB had a greater propensity to survive, a finding that has since proven to be a major mechanism of chemoresistance.^31^^,^^32^^,^^33^ Whether related to chemotherapy or cytokines such as TNF, which independently induce cell death, the last 25 years have produced a myriad of publications that have consistently shown that deletion of NF-κB activity in tumor cells accentuates stress-induced killing.^57^ As anticipated, our data recapitulated this dependence on NF-κB in a panel of epithelial and hematopoietic cell lines; however, we were unable to demonstrate similar dependence with FP and FN RMS tumor cells. Likewise, we previously showed *in vivo* that NF-κB signaling activity is required to suppress the differentiation of RMS,^27^ but this block is not sufficient to inhibit the development and progression of RMS.^58^ This leads us to conclude that unlike most solid and hematopoietic tumors that require NF-κB to overcome stress-induced killing, RMS represents a unique tumor type where the survival activity of NF-κB appears largely dispensable.

Our findings show that one mechanism that RMS tumor cells use to overcome their dependence on NF-κB is related to their partially differentiated phenotype. This was demonstrated in cultured myoblasts, where our data revealed that undifferentiated muscle cells lacking NF-κB were sensitive to stress, while those differentiated to myotubes were resistant to a similar stress. This was then confirmed when we reprogrammed both RMS cells and tumors with Yamanaka factors and showed that dedifferentiation caused their stress-induced death. Our findings are consistent with recent single cell and single nuclei RNA sequencing results, which revealed that clinically aggressive FP tumors correlate with a more differentiated phenotype.^24^ Still, to this point, what promotes RMS tumor cells to adopt this differentiation phenotype had not been determined.

In our study, we speculated that such a regulation could involve MRFs, since these factors are essential in establishing differentiation of muscle progenitors, and at least two of these MRFs, MyoD and myogenin, have been shown to be essential for post-natal muscle regeneration and development, respectively.^59^^,^^60^ In addition, both MyoD and myogenin are used as diagnostic markers of RMS.^16^^,^^17^ Our *in vitro* findings clearly showed that MyoD protects RMS tumor cells from stress to mediate their survival, results that were extrapolated to an *in vivo* setting. However, our results could not demonstrate a similar level of protection by myogenin, or to that matter, any of the other MRFs or MEF2 proteins. These data are well supported by results from the Broad Institute’s DepMap and the R2 Genomics Analysis and Visualization Platform. In both cases, among the MRFs and MEF2s tested, MyoD was identified as the most essential gene in RMS cell lines and displayed the strongest correlation with a negative patient outcome, respectively, which is consistent with earlier findings.^61^^,^^62^ Together, these data support that MyoD regulates a partial myogenic differentiation phenotype in RMS tumor cells, which serves as a survival mechanism against stress. Furthermore, we propose that this role of MyoD might be one reason why RMS tumors overcome dependence on NF-κB’s pro-survival activity.

One additional avenue of investigation that we pursued in this study was to determine the mechanism by which MyoD regulated the differentiation phenotype of RMS tumor cells. Through an *in silico* analysis of MyoD ChIP-seq datasets, we were surprised to discover that aside from established binding of MyoD on differentiation genes, such as MyHC and TnnT,^63^ whose expression in RMS tumor cells we had shown to be dependent on MyoD, enrichment peaks were also significantly displayed on stemness genes. We hypothesized that MyoD functioned in repressing these stem genes to thereby promote a differentiation phenotype in RMS tumor cells. However, we were unable to experimentally support this hypothesis. We therefore undertook an alternative line of investigation that led us to uncover a novel regulatory pathway by which MyoD represses the expression of death related genes. Our data suggest that repression of death genes occurs through an epigenetic mechanism via CpG methylation, mediated indirectly through MyoD activation of DNMT gene expression. A targeted CRISPR screen identified the gene CYLD,^54^^,^^56^ as a downstream target of MyoD. Results showed that the CYLD gene is repressed in RMS cells by DNMT activity and in addition is methylated at distinct CpG sites downstream of its promoter in a MyoD dependent manner. Although the functional relevance of CpG sites in the CYLD gene remain to be defined, knocking down CYLD rescued cell death in RMS tumor cells in response to TNF and use of a RIPK inhibitor supported that CYLD functions as a tumor suppressor in RMS through its established necroptotic signaling pathway.^54^ Collectively, these data provide fresh insight into the pathogenesis of RMS, by implicating MyoD as a direct regulator of DNMTs to mediate repression of CYLD, which in turn enhances survival of RMS. We suspect that future CRISPR screens will reveal additional unique death genes repressed by MyoD and DNMTs that lead to RMS survival under chemotherapy or other stress conditions.

Cumulatively, our findings are in line with a previous report that MyoD functions in RMS as an oncogene,^64^ rather than as a tumor suppressor as it has long been suggested. A standing paradigm in the RMS field has been that MyoD is impaired in regulating myogenesis, and that rescuing MyoD to promote differentiation might be an efficacious therapeutic strategy to combat this childhood cancer.^22^^,^^23^ Based on our current results, we suspect that such a strategy would be effective in inducing growth arrest, but in promoting this static condition would also allow tumor cells a survival advantage to overcome stress conditions imposed by chemotherapy or other treatment regimens, thereby potentially worsening patient outcome.

### Limitations of the study

Our functional data indicate that the effects of MyoD loss on cell survival in RH30 and RD cells were significantly amplified by concurrent suppression of NF-κB activity. As such, the relative contribution of MyoD to survival when NF-κB signaling is intact remains unresolved and will need to be further explored. In addition, the reliance on TNF in our CRISPR screen may have limited the identification of genes involved in more generalized stress responses. Future screens exposed to diverse stress conditions are needed to yield a more complete picture of MyoD-regulated survival networks. Lastly, our study did not define the mechanism by which MyoD regulates DNA methylation at the CYLD locus. A follow up study will determine whether MyoD recruits DNMTs or functions through intermediate chromatin remodeling pathways.

## Resource availability

### Lead contact

Further information and requests for resources and reagents should be directed to and will be fulfilled by the lead contact, Denis C. Guttridge (guttridg@musc.edu).

### Materials availability

Reagents generated in this study will be made available from the lead contact upon request.

### Data and code availability

• ATAC-seq and microarray raw datasets and analyzed data generated for this manuscript can be found on the Gene Expression Omnibus (GEO GSE224179).
• Original code for data analysis code has been deposited at Zenodo and is available at https://doi.org/10.5281/zenodo.15747354.
• Any additional information required to reanalyze the data reported in this paper is available from the lead contact upon request.

## Acknowledgments

We thank V. Spadafora and S. Miller of the Guttridge laboratory for their feedback and discussions throughout the course of this study. We are also grateful to members of the Roberts laboratory for their advice in bioinformatics and The Ohio State University Sarcoma Research Laboratory for their support and guidance during the early phases of this project. We are also extremely thankful to K. Kelly and C. Plass from the German Cancer Research Center and A. De Cubas from MUSC for their collective assistance in analyzing the CYLD gene for methylation. Support for this study was provided by NIH/NCI grants R01 CA235074 to D.C.G., R.D.R., and D.J.W., P01 CA163995 to P.J.H., C.A.L., D.C.G., R.D.R., D.J.W., and P.L., F30 CA265071 to A.R.O. and an 10.13039/100006108NIH NCATS award TL1 TR001451 to A.R.O.

## Author contributions

A.R.O., D.J.W., P.L., B.R.P., A.-A.U., S.S., P.Y.Y., E.E.T., E.M.H., C.J.M., B.K.K., J.H., C.A.L., P.J.H., and D.C.G. conceptualized, designed, and performed the experiments. A.R.O., D.J.W., B.R.P., S.S., M.A.A., R.D.R., and D.C.G. were involved in the methodology and A.R.O., S.S., P.Y.Y., and R.D.R. performed the bioinformatics and statistical analysis. A.R.O., P.Y.Y., B.R.P., and P.L. validated the data. D.C.G. planned and outlined the manuscript and the original draft was written by A.R.O. and D.C.G., while editing was performed by all the authors. A.R.O., D.J.W., and D.C.G. led the supervision and D.C.G. the project administration. A.R.O., E.E.T., C.A.L., P.J.H., R.D.R., and D.C.G. were involved in acquiring the funding to support the work.

## Declaration of interests

The authors declare no competing conflicts of interests.

## STAR★Methods

### Key resources table

**Table:** 

REAGENT or RESOURCE	SOURCE	IDENTIFIER
**Antibodies**		

Anti-alphaTubulin (mouse)	Thermo Fisher Scientific	Cat# A11126, RRID:AB_2534135
Anti-Mouse IgG (H + L) Antibody, HRP Conjugated	Promega	Cat# W4021, RRID:AB_430834
Anti-Rabbit IgG (H + L) Antibody, HRP Conjugated	Promega	Cat# W4011, RRID:AB_430833
Anti-IkappaB-alpha (rabbit)	Cell Signaling	Cat# 9242, RRID:AB_331623
Anti-MEF2C (rabbit)	Abcam	Cat# ab211493, RRID:AB_2864417
Anti-MEF2D (mouse)	Santa Cruz Biotechnology	Cat# sc-271153, RRID:AB_10614669
Anti-Myf6(MRF4) (mouse)	Santa Cruz Biotechnology	Cat# sc-514379
Anti-Myf-5 (mouse)	Santa Cruz Biotechnology	Cat# sc-518039
Anti-MYHC (Skeletal, Slow) (mouse)	Sigma-Aldrich	Cat# M8421, RRID:AB_477248
Anti-MyoD (mouse)	Santa Cruz Biotechnology	Cat# sc-377460, RRID:AB_2813894
Anti-MyoD (rabbit)	Abcam	Cat# ab133627, RRID:AB_2890928
Anti-myogenin (mouse)	Cell Marque	Cat# 296M-18, RRID:AB_1158682
Anti-myogenin (mouse)	Santa Cruz Biotechnology	Cat# sc-52903, RRID:AB_784707
Normal Mouse IgG	Millipore	Cat# CS200621
Anti-NFkappaB (p65) (rabbit)	Santa Cruz Biotechnology	Cat# sc-109, RRID:AB_632039
PE anti-mouse F4/80	BioLegend	Cat# 157303, RRID:AB_2832546
Anti-CYLD (rabbit)	Cell Signaling Technology	Cat# 4495, RRID:AB_10557111
Anti-RIPK1 (rabbit)	Thermo Fisher Scientific	Cat# PA5-20811, RRID:AB_11154790
Anti-Phospho-RIPK1 (mouse)	Proteintech	Cat# 66854-1-Ig, RRID:AB_2882194
Anti-Troponin T (mouse)	Sigma-Aldrich	Cat# SAB4200717, RRID:AB_2892079

**Bacterial and virus strains**		

Subcloning Efficiency DH5α competent cells	Thermo Fisher Scientific	Cat# 18265017
One shot Topo10 competent cells	Thermo Fisher Scientific	Cat# K457502

**Chemicals, peptides, and recombinant proteins**		

DTT	Invitrogen	Cat# Y00147
100 mM dNTP Set	Invitrogen	Cat# 10297-018
45 μm SFCA membrane filter	Thermo Fisher Scientific	Cat# 723-254
5X First Strand Buffer	Invitrogen	Cat# Y02321
Acrylamide	Life Science Products	Cat# EC-890-540
Actinomycin D	Sellecheck	Cat# S8964
Ammonium Persulfate	Sigma Aldrich	Cat# A3678-100G
APC Annexin V	BioLegend	640941
Blasticidin	GIBCO	Cat# A1113903
Bovine Serum Albumin	Thermo Fisher Scientific	Cat# BP9700100
Camptothecin	Selleckchem	Cat# S1288
Comassie Plus - The Better Bradford Assay Reagent	Thermo Fisher Scientific	Cat# 23238
CSF-1	R&D Systems	Cat# 216-MC-010/CF
D-Luciferin	Invitrogen	Cat# L2916
Decitabine	Sigma-Aldrich	Cat# A3656
DMEM	Corning	Cat# 10-017-CV
DNeasy Blood & Tissue Kit	Qiagen	Cat# 69504
Doxorubicin	Sigma-Aldrich	Cat# D1515
Dynabeads Protein G	Invitrogen	Cat# 10004D
ECL Prime Lumina Enhancer Solution	Cytiva	Cat# RPN2232V1/2
Esp3I Restriction Enzyme	NEB	Cat# R0734L
Etoposide	Selleckchem	Cat# S1225
FASTStart Universal SYBR Green Master (ROX)	Roche	Cat# 4913914001
FITC Annexin V	BioLegend	Cat# 51-65874X
FITC BrDU	BD Biosciences	Cat# 559619
Formaldehyde	Fisher	Cat# BP531-25
G418	Corning	Cat# 61-234-RG
Glycine	Sigma Aldrich	Cat# G7126-5KG
GSK’963	Sellecheck	Cat# S8642
Herring Sperm DNA	Invitrogen	Cat# 15634-017
High Pure RNA Isolation Kit	Roche	Cat# 11828665001
hygromycin	Thermo Scientific	Cat# 10687010
IFNγ	R&D Systems	Cat# 285-IF-100/CF
Lipofectamine 2000	Thermo Fisher Scientific	Cat# 11668027
Lipofectamine RNAiMax	Thermo Fisher Scientific	Cat# 13778075
live/dead fixable near-IR dead cell stain kit	Thermo Fisher Scientific	Cat# L10119
LPS	Sigma-Aldrich	Cat# 916374
M-MLVRT	Invitrogen	Cat# 28025-013
M-PER™ Mammalian Protein Extraction Reagent	Thermo Scientific	Cat# 78501
Mitomycin C	Sigma-Aldrich	Cat# M5353
NaVO3	Sigma-Aldrich	Cat# 508605
Nitrocellulose blotting membrane	GE Healthcare Life science	Cat# 10600002
Nonfat dried milk powder	AppliChem	Cat# A0830
PE Annexin V	BioLegend	Cat# 640947
PMSF	Thermo Fisher Scientific	Cat# 36978
ProFection Mammalian Transfection System---Calcium Phosphate, 40 reactions, Promega	Promega	Cat# PAE1200
Protease Inhibitor Cocktail	Abcam	Cat# ab201111
puromycin	Gibco	Cat# A1113803
PVDF Membrane	Millipore	Cat# IPVH00010
RPMI	Corning	Cat# 10-040-CV
SDS	Thermo Fisher Scientific	Cat# 28312
Sodium Nitroprusside (SNP)	Sigma-Aldrich	Cat# 71778
Tris (hydroxymethyl)aminomethane	Thermo Fisher Scientific	Cat# J65594.A7
TRIzol	Invitrogen	Cat# 15596026
Tumor Necrosis Factor-α, mouse (mTNF-α)	Roche	Cat# 11271156001
Tumor Necrosis Factor-α, mouse (mTNF-α)	Roche	Cat# 11271156001
Vincristine	Tocris	Cat# 1257
X-gal	Thermo Fisher Scientific	Cat# R0404
IPTG	Sigma-Aldrich	Cat# 16758-5G
Platinum II Taq hot_start DNA polymerase	Thermo Fisher Scientific	Cat# 14966001
Platinum II Taq high fidelity DNA polymerase	Thermo Fisher Scientific	Cat# 11304011
Guanidine thiocyanate	Sigma-Aldrich	Cat# G9277-250G
RNase Inhibitor	New England Biolab	Cat# M0314S
SuperScript IV Reverse Transcriptase	Thermo Fisher Scientific	Cat# 18090200

**Critical commercial assays**		

Caspase-Glo® 3/7 Assay System	Promega	Cat# G8090
Qiagen DNA Blood Kit	Qiagen	Cat#51104
Epitect Bisulfite Kit	Qiagen	Cat#59104
Qiagen Gel Extraction Kit	Qiagen	Cat#28704
Qiagen Plasmid Mini Prep Kit	Qiagen	Cat#27106
Topo TA Cloning Kit	Thermo Fisher Scientific	Cat# K457502

**Deposited data**		

Raw and analyzed data	This paper	GEO: GSE224179
Human reference genome NCBI build 38, GRCh38	Genome Reference Consortium	http://www.ncbi.nlm.nih.gov/projects/genome/assembly/grc/human/
MyoD ChIP-seq RD cells	MacQuarrie et al.^48^	GEO: GSE50413
MyoD ChIP-seq RH4 cells	Gryder et al.^47^	GEO: GSE83726

**Experimental models: Cell lines**		

697		RRID:CVCL_0079
A549	ATCC	Cat# CCL-185, RRID:CVCL_0023
C2C12	ATCC	Cat# CRL-1772, RRID:CVCL_0188
DU145	ATCC	Cat# HTB-81, RRID:CVCL_0105
hSk	Lonza	Cat# CC-2580
Jurkat		RRID:CVCL_0065
LNCAP		Cat# CRL-1740, RRID:CVCL_1379
MDA231	ATCC	Cat# HTB-26, RRID:CVCL_0062
Panc2	ATCC	Cat# CRL-2553, RRID:CVCL_1633
RD	ATCC	Cat#CCL-136
RD2		NA
RH3		RRID:CVCL_L415
RH5		RRID:CVCL_5917
RH18		RRID:CVCL_1659
RH30		RRID:CVCL_0041
RH4		RRID:CVCL_5916
RH36		RRID:CVCL_M599

**Experimental models: Organisms/strains**		

C57BL/6J mice	JAX	RRID:IMSR_JAX:000664
SCID^ICR^ mice	Taconic	RRID:IMSR_TAC:icrsc

**Oligonucleotides**		

NF-kB EMSA Probe, see Table S3	Guttridge et al.^65^	NA
oligo (dT)	This paper	NA
Oligo(dT)VN	This paper	NA
Primers for qPCR, see Table S3	This paper	NA
Primers for ChIP, see Table S3	This paper	NA
Primers for Bisulfite PCR, see Table S3	This paper	NA
p65 siRNA (P1D1), see Table S3	This paper	NA
GFP siRNA, see Table S3	This paper	NA

**Recombinant DNA**		

GIPZ Lentiviral MEF2C shRNA	Horizon/Dharmacon	Cat# V2LHS_151683, V2LHS_151686, V3LHS_370639, V3LHS_370640, V3LHS_370642
GIPZ Lentiviral MEF2D shRNA	Horizon/Dharmacon	Cat# V3LHS_410959, V3LHS_376783, V3LHS_410961
GIPZ Lentiviral MYF5 shRNA	Horizon/Dharmacon	Cat# V2LHS_62144, V3LHS_340745, V3LHS_340746
GIPZ Lentiviral MYF6 (MRF4) shRNA	Horizon/Dharmacon	Cat# V2LHS_152060, V3LHS_395481, V3LHS_395482
GIPZ Lentiviral MYOD1 shRNA	Horizon/Dharmacon	Cat# V3LHS_370537, V3LHS_409996, V2LHS_15209
GIPZ Lentiviral MYOG (myogenin) shRNA	Horizon/Dharmacon	Cat# V2LHS_152118, V3LHS_398736, V3LHS_398737, V3LHS_398740
GIPZ Non-silencing Lentiviral shRNA Control	Horizon/Dharmacon	Cat# RHS4346
lenti-sgRNA-neo	Addgene	RRID:Addgene_104992
lenti-sgRNA-puro	Addgene	RRID:Addgene_104990
lentiCas9-Blast	Addgene	RRID:Addgene_52962
p1242 3x-KB-L	Addgene	RRID:Addgene_26699
pBABE-hygro	Addgene	RRID:Addgene_1765
pBABE-MyoD-L122R-puro	This paper	NA
pBABE-MyoD-wt-puro	Guttridge et al.^66^	NA
pBABE-puro	Addgene	RRID:Addgene_1764
pMXs-Klf4	Addgene	RRID:Addgene_17219
pMXs-Myc	Addgene	RRID:Addgene_17220
pMXs-Oct3/4	Addgene	RRID:Addgene_17217
pMXs-Sox2	Addgene	RRID:Addgene_17218
pX330	Addgene	RRID:Addgene_140610

**Software and algorithms**		

Adobe Photoshop and Illustrator CS6	Adobe	NA
Venny 2.1.0	https://bioinfogp.cnb.csic.es/tools/venny/	RRID:SCR_016561
ToppGene Suite	https://toppgene.cchmc.org/	RRID:SCR_005726
Rsubread	Liao et al.^67^	RRID:SCR_016945
Rsamtools	Li et al.^68^	NA
MACS2	Zhang et al.^69^	RRID:SCR_013291
GenomicAlignment	Lawrence et al.^70^	NA
Herper	10.18129/B9.bioc.Herper	NA
ChIPseeker	Yu et al.^71^	RRID:SCR_021322
DESeq2	Love et al.^72^	RRID:SCR_015687
oligo	Carvalho et al.^73^	RRID:SCR_015729
affycoretools	https://doi.org/10.18129/B9.bioc.affycoretools	NA
Limma	Smyth et al.^74^	RRID:SCR_010943
GraphPad Prism	https://www.graphpad.com/	NA
The Cancer Genome Atlas (TCGA)	NA	RRID:SCR_003193
UCSC Xena	https://xenabrowser.net/	RRID:SCR_018938
Cancer Dependency Map Portal	https://depmap.org/portal/achilles/	RRID:SCR_017655
FlowJo	https://www.flowjo.com/	RRID:SCR_008520
Integrative Genome Viewer	https://software.broadinstitute.org/software/igv/	RRID:SCR_011793
Gene Expression Omnibus	https://www.ncbi.nlm.nih.gov/geo/	RRID:SCR_005012
C++		NA
Python/Biopython		NA
Cancer Cell Line Encyclopedia dataset	https://sites.broadinstitute.org/ccle/	NA
R2 Genomics Analysis and Visualization Platform	https://hgserver1.amc.nl/cgi-bin/r2/main.cgi?open_page=login	NA
Clusteral Omega	https://www.ebi.ac.uk/jdispatcher/msa/clustalo	NA

### Experimental model and subject details

#### Animals

This study was performed in accordance with ARRIVE guidelines. All animal experiments were approved by The Ohio State University Institutional Animal Care and Use Committee under protocol 2010A00000177 and the Medical University of South Carolina by the Animal Research Multiple Project Assurance (MPA# MUSC—D16-00268 (A3428-01)). All animals were housed in the animal facility at the MUSC with constant temperature and humidity and fed a standard diet *ad libitum* with free access to water. All procedures and mouse treatments in conforming with appropriate regulation and guideline were reviewed and approved by the Institutional Animal Care and Use Committee (IACUC) under our animal protocol, IACUC-2024–01711, at MUSC. Female 10–12 -week-old SCID^ICR^ mice (RRID:IMSR_TAC:icrsc) weighing 22–30 grams were randomly divided into control or experimental groups prior to experiments.

#### Cell lines and culture conditions

The cell lines A549 (Cat# CCL-185, RRID:CVCL_0023), Panc2 (Cat# CRL-2553, RRID:CVCL_1633), DU145 (Cat# HTB-81, RRID:CVCL_0105), LNCAP (Cat# CRL-1740, RRID:CVCL_1379), MDA231 (Cat#HTB-26, RRID:CVCL_0062), C2C12 (Cat# CRL-1772, RRID:CVCL_0188) and 293T (CRL-3216) were purchased from the American Type Culture Collection (ATCC) and cultured in DMEM (Corning Cat# 10-017-CV) supplemented with 10% heat inactivated FBS (R&D systems Cat# S11150H) and 1% Penicillin-Streptomycin (Corning Cat# 30-001-CI). Rhabdomyosarcoma cell lines RH30 (male, RRID:CVCL_0041), RD (female, RRID:CVCL_1649), RH18 (RRID:CVCL_1659), RH5 (RRID:CVCL_5917), RH3 (RRID:CVCL_L415), RH36 (RRID:CVCL_M599), RD2 and leukemia cell lines Jurkat (RRID:CVCL_0065) and 697 (RRID:CVCL_0079) were cultured in RPMI (Corning Cat# 10-040-CV) with 10% heat inactivated FBS and 1% antibiotics. C2C12 mouse myoblasts were differentiated in DMEM containing 2% horse serum (Corning). Human skeletal muscle myoblasts (hSk, Lonza Cat# CC-2580) were cultured in growth media per the supplier’s instructions. All cells were cultured in a humidified tissue culture hood at 37 with 5% CO_2_.

### Method details

#### Materials

Chemicals for cell treatment included TNF (Roche, 11271156001), doxorubicin (DOX, Sigma-Aldrich Cat# D1515), etoposide (ETOP, Selleckchem Cat# S1225), camptothecin (CPT, Selleckchem Cat# S1288), vincristine (VIN, Tocris Cat# 1257), actinomycin D (ACTD, Selleckchem Cat# S8964), decitabine (Sigma Cat# A3656). Reagents for specific assays are listed below.

#### Virus preparation and transduction of cell lines

Retrovirus expression plasmids were generated using with pBABE vector system. IκBα-Super Repressor (SR) was subcloned into both pBABE-puro (RRID:Addgene_1764) and pBABE-hygro (RRID:Addgene_1765) plasmids. The human MyoD coding sequence, derived from pEMC11s, was subcloned into pBABE-puro with an FLAG tag and was subjected to site-directed mutagenesis to produce pBABE-puro.MYOD.L122R.^66^ The Yamanaka factor retroviral expression vectors pMXs-hKLF4 (RRID:Addgene_17219), pMXs-hOCT3/4 (RRID:Addgene_17217), pMXs-hc-MYC (RRID:Addgene_17220), and pMXs-hSOX2 (RRID:Addgene_17218) were purchased from Addgene for cellular reprogramming experiments. All viruses were prepared by calcium phosphate mediated transfection kit (Promega Cat# PAE1200) with 293T cells using a mixture of standard retroviral or lentiviral packaging plasmids. Viruses were harvested from supernatant 48 h post transfection and purified by centrifugation 2,000 × *g* for 10 min at 4*°*C, followed by filtration with a 45 μm SFCA membrane filter (Thermo Fisher Scientific Cat# 723–2545). Except otherwise indicated, approximately 5-10pfu/cell of viruses in a total volume of 4 mL/100 mm dished containing 4 mg/mL of polybrene (Sigma-Aldrich Cat# TR-1003) was utilized to transduce cells for 16 h. Infected cells were selected using antibiotics at empirically decided doses [puromycin (Gibco Cat# A1113803), hygromycin (Thermo Fisher Scientific 10687010), neomycin/G418 (Corning Cat# 61-234-RG), or blasticidin (Gibco Cat# A1113903)].

#### siRNA and sh-RNA knockdown

A self-designed small interfering RNA (siRNA) scrambled and siRNA targeting the p65/RelA subunit of NF-κB were transfected into target cells using Lipofectamine 2000 (Thermo Fisher Scientific Cat# 11668027) according to the manufacturer’s recommendations (Table S3). Small hairpin RNA (sh-RNA) lentiviral plasmids were purchased from Horizon/Dharmacon to target Myogenic Regulatory Factors (MRFs) or Myocyte Enhancing Factors (MEFs). These included MyoD (Cat# V3LHS_370537, V3LHS_409996, V2LHS_15209), Myf5 (V2LHS_62144, V3LHS_340745, V3LHS_340746), myogenin (Cat# V2LHS_152118, V3LHS_398736, V3LHS_398737, V3LHS_398740), MRF4 (Cat# V2LHS_152060, V3LHS_395481, V3LHS_395482), MEF2C (Cat# V2LHS_151683, V2LHS_151686, V3LHS_370639, V3LHS_370640, V3LHS_370642), MEF2D (Cat# V3LHS_410959, V3LHS_376783, V3LHS_410961). A scramble sh-RNA (Horizon Cat# RHS4346) was used as a control. These sh-RNAs were either transfected into target cells using Lipofectamine 2000 or prepared into lentiviral particles and infected into cells followed by antibiotic selection. Degradation of target RNA and loss of protein were quantified by qRT-PCR or Western blot analysis, respectively.

#### CRISPR/Cas9 gene editing

Small guide RNA (sgRNA) coding sequences were designed with CHOPCHOP (https://chopchop.cbu.uib.no/). After removing palindromic DNA sequence NGG, adequate sequences were added in front and after the coding sequence pairs to promote ligation into vectors and initiation of transcription. Oligos coding for sgRNA were synthesized by IDT DNA (https://www.idtdna.com/). In a total of 5 p.m., each forward and reverse synthesized oligos were annealed into double strand DNA in 50 μL solution containing 1× PCR buffer (Thermo Fisher, #14966001) on a thermocycler with a program as follows: 95°C (5 min) → 0.2°C/s → 85°C (incubate 30 s) → 0.1°C/s → 80°C (3 min) → 0.1°C/s → 75°C (4 min) → 0.1°C/s → 60°C (5 min) → 0.1°C/s → 37°C (60 min) → hold at 10°C. After annealing, 1 μL of annealed oligos were utilized for ligation into pX330 (RRID:Addgene_140610) or lenti-sgRNA (puro RRID:Addgene_104990 or *neo* RRID:Addgene_104992) vectors for expressing gene specific sgRNAs for Cas9 mediated gene knockout. For gene deletion with pX330, single cell clones were selected after lipofectamine mediated gene transfection. For lentivirus CRISPR technology, Cas9 enzyme was expressed on a separate lentiCas9-Blast construct (RRID:Addgene_52962) and infected cells were directly checked for gene knockout and used for experiments after antibiotic selection. An sgRNA designed from a random genomic DNA sequence without any similarities to human genome was utilized as a scramble control. See Table S3 for sequences of sgRNA insert and their annealing oligos with adaptor sequences. Lentiviruses containing sgRNA targeting genes or Cas9 enzyme were prepared as mentioned above and co-infected in respective cell lines. Loss of protein expression was confirmed by Western blot analysis post antibiotic selection.

#### CRISPR sgRNA library screen

A custom library containing two sgRNAs for each gene were synthesized to target 25 genes found regulated by MyoD (Table S3). After ligation into lenti-virus sgRNA expression vectors, equal amount of plasmid DNA was mixed and packaged into lentivirus particles as outlined above. RH30-SR MyoD^Δ^ cells stably expressing the Cas9 enzyme were infected with the lentivirus library with undiluted virus or at a level of 0.5 particles per cell, determined empirically. Cells were maintained under antibiotic selection. Media was changed without selection 24 h before TNF treatment. Cells were treated with either 10 ng/mL TNF for 24 or 48 h. After treatment, cells were re-cultured in selection media and were allowed to recover for 72 h. This process was repeated for a total of 4 cycles in the 24 h treatment scheme or 2 cycles in the 48 h treatment regimen. A control plate of infected cells was passaged throughout each experiment. Genomic DNA was isolated with the DNeasy Blood & Tissue Kit (Qiagen Cat# 69504). The amount of sgRNA was quantified by qPCR of genomic DNA, standardizing the TNF treated samples to the untreated control.

#### Electrophoretic mobility shift assay (EMSA)

EMSAs were performed to assess for NF-κB DNA binding activity as previously described.^65^^,^^75^

#### Immunoblotting and antibodies

Cell pellets were lysed with RIPA buffer containing protease inhibitor cocktail (Abcam Cat# ab201111) and phosphatase inhibitors NaVO_3_ (1.25 mM, Sigma-Aldrich Cat# 5.08605) and PMSF (2 mM, Thermo Fisher Scientific Cat# 36978). Protein concentration was measured with Bradford Assay Reagent (Thermo Fisher Scientific Cat# 23238). A total of 20 μg of protein sample was denatured in 4X SDS sample loading buffer at 95°C for 5 min. Lysate was resolved in 10–12% SDS-PAGE gels and transferred to nitrocellulose (GE Healthcare Cat# 10600002) or activated PVDF membrane (Millipore Cat# IPVH00010). Membranes were blocked in TBST (TBS, 0.1% Tween 20) buffer containing 5% non-fat milk (AppliChem Cat# A0830) while being agitated for 1 h at room temperature or 4°C overnight. Primary antibodies were incubated in 5% non-fat milk in TBST with NaN_3_ (0.02%). Membranes were washed with TBST 3–5 times for a minimum duration of 5 min before proceeding to incubation with HRP conjugated secondary antibody in 2.5% non-fat milk in TBST. Membranes were washed again in TBST prior to incubation with ECL (Cytiva Cat# RPN2232V1/2). Blots were imaged on a ChemiDoc (Biorad Cat# 12003153). Blots were stripped with stripping buffer (2% SDS, 62.5 mM Tris pH 6.8, 100 mM 2-mercaptoethanol) for re-probing. Antibodies used in this study included, IκBα (Cell Signaling Technology Cat# 9242, RRID:AB_331623), α-tubulin (Thermo Fisher Scientific Cat# A11126, RRID:AB_2534135), p65/RelA (Santa Cruz Biotechnology Cat# sc-109, RRID:AB_632039), MyoD (Santa Cruz Biotechnology Cat# sc-377460, RRID:AB_2813894), Myf5 (Santa Cruz Biotechnology Cat# sc-518039), myogenin (Santa Cruz Biotechnology Cat# sc-52903, RRID:AB_784707), MRF4 (Santa Cruz Biotechnology sc-514379), MEF2C (Abcam Cat# ab211493, RRID:AB_2864417), MEF2D (Santa Cruz Biotechnology Cat# sc-271153, RRID:AB_10614669), MYHC (Sigma-Aldrich Cat# M8421, RRID:AB_477248), TnnT (Sigma-Aldrich Cat# SAB4200717, RRID:AB_2892079), CYLD (Cell Signaling Technology Cat# 4495, RRID:AB_10557111), *p*-RIPK1 (Proteintech Cat# 66854-1-Ig, RRID:AB_2882194), RIPK1 (Thermo Fisher Scientific Cat# PA5-20811, RRID:AB_11154790), DNMT1 (Novus Cat# NB100-56519, RRID:AB_838131), DNMT3A (Santa Cruz Biotechnology Cat# sc-365769, RRID:AB_10844010), DNMT3B (Cell Signaling Technology Cat# 67259, RRID:AB_2799723), and HRP conjugated secondary antibodies anti-mouse IgG (Promega Cat# W4021, RRID:AB_430834) and anti-rabbit IgG (Promega Cat# W4011, RRID:AB_430833).

#### Semi-quantitative PCR (qPCR)

For qPCR Analysis, total RNA was isolated from either cell pellets or tumor samples using the High Pure RNA Isolation Kit (Roche, Cat# 11828665001) or TRIzol Reagent (Invitrogen, Cat# 15596026). cDNA synthesis was performed using 3 μg of total RNA, oligo(dT) primers, and M-MLV reverse transcriptase (Invitrogen, Cat# 28025-013).

For single-cell qPCR, RH30 cells were harvested by trypsinization, washed with PBS, and resuspended at 1 million cells per mL in PBS containing 0.1% BSA and 1 μL/mL of cell survival dye (Invitrogen, Cat# L34975). RH30 cells were sorted into 96-well plates at 1 cell/well, with each well containing 2 μL of 50 mM guanidine thiocyanate.

#### Reverse transcription (RT) for single-cell qPCR

Sorted cells were reverse transcribed into cDNA using SuperScript IV Reverse Transcriptase (Invitrogen, Cat# 18090200) with the following reaction setup in each well.

(1) Add:
  a. OligodTVN (2 pmol/μL) – 1 μL.
  b. 10 mM dNTP mix – 1 μL.
(2) Incubate at 65°C for 10 min, then cool to room temperature for 5 min.
(3) Add 6 μL of reverse transcription solution, containing:
  a. 1× First-Strand Buffer.
  b. 5 mM DTT.
  c. 20 U RNase Inhibitor (NEB, Cat# M0314S).
  d. 40 U SuperScript IV Reverse Transcriptase.
(4) Incubate at:
  a. 50°C for 30 min.
  b. 55°C for 15 min.
  c. 80°C for 10 min (to inactivate the enzyme).

#### Pre-amplification and qPCR

From each well, 4 μL of cDNA was pre-amplified using Hot Start Taq Polymerase (Invitrogen/Thermo Fisher, Cat# 14966001) with primers for MyoD, Myogenin, TNNT, MyH1, and Pax7 at 0.2 pmol/μL each, for 11 cycles. The amplified cDNA was then diluted to 100 μL, and 2 μL was used for further qPCR amplification. For GAPDH qPCR, 2 μL of reverse-transcribed cDNA was directly used. All qPCR reactions were performed using the QuantStudio 3 Real-Time PCR System (Applied Biosystems, Cat# A28567) with FASTStart Universal SYBR Green Master Mix (Roche, Cat# 4913914001). Oligonucleotides were synthesized by IDT DNA Technologies. A complete list of sequences is provided in Table S3. GAPDH was used as an internal control.

#### ChIP assays

ChIP assays were done following standard protocols. In brief, log phase growing RH30 cells were fixed in 1% formaldehyde (Fisher Cat# BP531-25) for 8 min. Formaldehyde was quenched with 125 mM Glycine while shaking for 5 min at room temperature. Cells were washed twice with phosphate buffered saline (PBS) and harvested by scraping. Cell pellets were lysed in LB3 ChIP lysis buffer (10 mM Tris-HCl pH 8.0, 1 mM EDTA, 100 mM NaCl, 0.5% N-lauroylsarcosine, 0.1% sodium deoxycholate), and sonicated with Bioruptor NGS (Diagenode Cat# UCD-600) at empirically optimized conditions. Sonicated cell lysates were centrifuged, and supernatants were precleared with protein G magnetic beads (Invitrogen Cat# 10004D) saturated with herring sperm DNA (75 ng/μL, Invitrogen Cat# 15634-017) and bovine serum albumin (0.1 μg/μL, Fisher Cat# BP9700100). Precleared lysates were precipitated with monoclonal mouse anti-human MyoD antibody (Santa Cruz Biotechnology Cat# sc-377460, RRID:AB_2813894) or mouse IgG (Millipore Cat# CS200261) bound to protein G magnetic beads (saturated with herring sperm DNA and bovine serum albumin). Precipitated DNA was de-crosslinked and subjected to qPCR as described above to amplify putative MyoD binding sites in RMS. A complete list of primer sequences is listed in Table S3.

#### Luciferase assays

Luciferase reporter assays were performed as previously described.^60^ Briefly, cells were transfected with NF-κB luciferase reporter derived from p1242 3x-κB-Luc (RRID:Addgene_26699) using Lipofectamine 2000 and the manufacturer’s instructions and seeded into 24 well plates. Cells were then treated with 5 ng/mL of TNF for 2 h, washed with PBS and lysed with M-PER Mammalian Protein Extraction Reagent (Thermo Fisher Scientific Cat# 78501) at room temperature while shaking for 5 min. Cell debris was cleared by centrifugation (16,000 × *g*, 4°C, 10 min) and supernatant was stored at −80°C. Luciferase activity was monitored in protein lysate using D-Luciferin (Invitrogen Cat# L2916) and a SpectraMax iD5 (Molecular Devices), and light units were recorded. Caspase activity of C2C12 cells following TNF was quantified by Caspase-Glo 3/7 Assay System (Promega Cat# G8090) via detection of luciferase activity due to caspase degradation of substrate, as described by the manufacturer’s protocol.

#### Fluorescence-activated cell sorting (FACS) analysis

FACS analysis for death assays was performed as previously described.^61^ Briefly, cells cultured in selection media were changed into selection free media 24 h prior to collection. Harvested cells were plated at 6 × 10^5^ cells/60 mm dish and cultured overnight before further treatments. All cells were harvested with trypsinization. Cell pellets were washed with PBS and with Annexin V staining buffer (0.01 M HEPES pH 7.4, 140 mM NaCl, 2.5 mM CaCl_2_). Cells were then stained with Annexin V (BioLengends Cat# PE: 640947, FITC: 51-65874X, APC: 640941) and live/dead fixable near-IR dead cell stain kit (Thermo Fisher Scientific Cat# L10119). Cells were then fixed with 3.7% formaldehyde in staining buffer and washed with PBS. FACS analysis of peritonium infiltrating macrophages was performed as previously described.^76^ In brief, 1 × 10^6^ RH30-SR or RH30-SR MyoD^Δ^ cells were injected into the peritoneal cavity of SCID mice. Five days after injection, mice were euthanized and the peritonium membrane exposed. Then 9 mL of DMEM plus 10% FBS were injected into the peritoneal cavity with a syringe attached with a 26G needle. After swirling for 30 s, cells were harvested with a paster pipette. Harvested cells were centrifuged and incubated on ice in 100 μL of PBS containing 5% BSA and 0.5 μg rat anti mouse CD16/CD32 (Biolegend Cat# 156604, RRID:AB_10766954) for 15 min and then 1 μL of PE anti-mouse F4/80 (BioLegend Cat# 157303, RRID:AB_2832546) was added and incubated for 30 min. After washing twice with PBS, cells were suspended into 1 mL PBS/0.5% BSA for quantification of F4/80 positive peritoneal macrophages in response to inoculation of RMS tumor cells as previously described.^76^ All Flow cytometry was performed with the BD LSR Fortessa or BD CytoFLEX LX cytometers. FCS files were processed using FlowJo software (RRID:SCR_008520).

#### Mitomycin C treatment

Mitomycin C (5 μg/mL, Sigma-Aldrich Cat# M5353) was added into C2C12 cell cultures at 75–80% confluency for 4 h with DMSO treatment used as a control. Treated cells were harvested by trypsinization and plated at 1 × 10^4^ cells/well in 24 well plates for cell counting. For TNF induced cell death analysis, Mitomycin C treated cells were further treated with TNF at 5 ng/mL overnight. Harvested cells were stained with Annexin V and fixable cell survival dye as described above for FACS analysis.

#### Cell cycle assays

Mitomycin C treated cells were pulsed with BrdU at 10 μM for 4–6 h and harvested by trypsinization. BrdU staining was performed using a BrdU detection kit from (BD Biosciences Cat# 559619) with a protocol suggested by the manufacturer. In brief, harvested cells were fixed for 30 min by adding ice-cold ethanol to 50%. Fixed cells were permeabilized with 2 M HCl, 0.5% Triton X-100 for 30 min. Cells were then washed once with 0.1 M sodium tetraborate pH 8.5 and twice with PBS containing 1%BSA, followed by staining with 10 μg/sample FITC conjugated anti-BrdU in PBS containing 0.5% Tween 20 and 1% BSA followed by 50 μg/mL 7-AAD for 30 min as recommended by manufacturer. Another 1 mL PBS was added into each tube and stained cells were analyzed by Fortessa X-20. Cell growth analysis on C2C12-SR cells with or without treatment of mitomycin was performed as previously described.^45^

#### Colony assay

Bottom agar (1%) or top agar (0.3%) was made by mixing 4% Noble Agar with DMEM containing 10% FBS at 50°C. None tissue culture treated 6 well plates (Fisher Scientific, cat# 07-000-646) were overlayed with 2 mL bottom agar and equilibrated to room temperature. Cells in culture media pre-warmed to 40°C were added into top agar at the same temperature in a 1:20 cell volume to top agar volume. Cell containing top agar solution (2 mL) were immediately overlayed on top of 6 wells plates with 2 mL bottom agar and equilibrated to room temperature. After cooling at rt for 5 min, these 6 well plates were transferred into 37°C humidified incubators with 5% CO2. Cells were cultured for 10–20 days and stained with 0.005% crystal violet in PBS for 1 h before colony counting.

#### Coculture assays

C57BL/6J (RRID:IMSR_JAX:000664) or C57BL/6J *TNF*^−/−^ (RRID:IMSR_JAX:003008) mice were euthanized at 8–10 weeks of age by CO2 inhalation. Both femurs of mice were isolated and bone marrow cells were flushed into 10 mL of full culture media (DMEM containing 10% Heat inactivated FBS, 1% antibiotics). Isolated cells were dispersed by repeated pipetting and filtered through 40 μM cell strainer to remove debris. RBC lysis was performed for 10 min at room temperature with 160 mM NH_4_Cl and halted with addition of 30 mL of PBS. Cells were washed with PBS and cultured overnight with full media in tissue culture dishes. Suspension cells were counted the following day and cultured at 3 × 10^6^ cells/100 mm dish in full culture media supplemented with 25 ng/mL CSF-1 (R&D Systems Cat# 216-MC-010/CF) for 5 days. For macrophage activation, LPS (50 ng/mL final, Sigma-Aldrich Cat# 916374) and IFNγ (2 ng/mL final, R&D Systems Cat# 285-IF-100/CF) were added to induce M1 polarization. Activated macrophages were harvested by trypsinization and cell scraping. Log phase growing RH30-SR or RH30-SR MyoD^Δ^ cells were harvested by trypsinization, and plated in 24 well plate for overnight. Next day, either wildtype or *TNF*^−/−^ bone marrow derived macrophages were applied in 20:1, macrophage:RH30 ratio in DMEM supplemented with heat-inactivated FBS. After 48 h, cells were trypsinized and surviving cells were counted by a trypan blue exclusion assay. Control conditions contained RH30-SR or RH30-SR MyoD^Δ^ cells without macrophages.

#### RMS patient data and DepMap portal

RMS patient dataset analyzed by the R2 Genomic Analysis and Visualization Platform (https://hgserver1.amc.nl/cgi-bin/r2/main.cgi?open_page=login) were utilized to perform Kaplan-Meier Survival analysis correlated to MyoD, myogenin, MRF4, MEF2C, and MEF2D expression. Survival data was limited to 5 years after diagnosis. Expression dot plots for all tumors from these patients in the R2 dataset were generated for MyoD, myogenin, MYHC (MYH1) and TNNT1. The Broad Institute’s Cancer Dependency Map Portal (RRID:SCR_017655) CRISPR dependency screen, Project Achilles (https://depmap.org/portal/achilles/), was used to determine the CHRONOS score, or dependency score as previously described,^41^^,^^42^ for MyoD, Myf5, Myf6/MRF4, myogenin, MEF2C, and MEF2D across all sarcoma cell lines.

#### Immunohistochemistry

Formalin-fixed paraffin embedded patient embryonal and alveolar rhabdomyosarcoma tumor samples were sectioned at 4 μm thickness. Immunoperoxidase staining for MYOD1 (Abcam Cat# ab133627, RRID:AB_2890928) and myogenin (Cell Marque Cat# 296M-18, RRID:AB_1158682) were scored following sample randomization, using previously published standards of 1+ to 4+ based on total percentage of positively stained nuclei. Stained sections were evaluated by a trained pathologist (MAA) and scores for each section were estimated as an average over the entire tumor area.

#### Xenograft tumor assays

RH30-SR cells were infected with Cas9 and either Vector control sgRNA or sgRNA targeting MyoD lentiviruses. To ensure complete ablation of MyoD, two independent clones of RH30-SR MyoD^Δ^, (CMD2 and CMD29) were selected for xenograft assays. A total of 3 × 10^6^ cells were injected subcutaneously into the flank of randomly selected 10–12 week-old female SCID^ICR^ mice (RRID:IMSR_TAC:icrsc, *n* = 7). Tumor diameter measurements were performed every other day with a digital caliper and volume was estimated through the formula π/6∗ (larger diameter)∗(shorter diameter). Kaplan-Meier survival curves were calculated for mice which were euthanized by reaching tumor endpoint criteria. For reprogramming experiments, mice were injected with RH30-SR cells and divided into equal groups when tumor volumes reached approximately 1 cm^3^ (*n* = 5). Tumors were directly injected with either a GFP control virus or retroviruses containing three Yamanaka factors. Mice were then treated with 1 mg/kg vincristine at 7 and 14 days post viral infection and tumor volumes were subsequently measured. Total RNA was isolated from the periphery of the tumor outside of the necrotic core and assayed for expression of MyoD, myogenin, Tnnt1, and Pax7 by qPCR. All procedures in mice were carried out according to protocols approved by the Institutional Animal Care and Use Committee at The Ohio State University and Medical University of South Carolina.

#### Microarray

Differential gene expression was performed by microarray analysis at The Ohio State University Comprehensive Cancer Center Genomics Core. The GeneChip Human Transcriptome Array 2.0 (Affymatrix Cat# 902162) is comprised of >6 million unique probes including coding and non-coding RNA transcripts, for a total of 67,000 human genes (transcript clusters). Differential expression analysis was carried out in R. Briefly, CEL files were opened with the oligo package (RRID:SCR_015729) and normalized using the RMA function.^73^ Expression set data was annotated with the Bioconductor packages affycoretools and pd.hta.2.0. Annotations were subsequently mapped to human genes symbols and ENTREZ IDs. The LIMMA R package (RRID:SCR_010943) was used for differential expression analysis between the three groups: RH30, RH30-SR, and RH30-SR MyoD^Δ^.^74^ To test the pairwise comparison of each condition, we used a standard LIMMA pipeline. First, we established a design matrix accounting for each group and fit linear models to the expression data. We then fit a contrast matrix to the data comparing all combinations of conditions. The t statistics were then calculated with the empirical Bayes method. Genes were filtered for a fold change >25% and an adjusted *p*-value <0.05 with the Benjamini-Hochberg correction. ToppGene Suite (https://toppgene.cchmc.org/, RRID:SCR_005726) was used for all Gene Ontology (GO) analyses. Raw sequences and analyzed files were deposited on the Gene Expression Omnibus (GEO, RRID:SCR_005012; GEO GSE224179).

#### ATAC-seq

Cells were trypsinized and frozen in 10% DMSO and 50% FBS containing media. Samples were sent to Quick Biology Inc. (Monrova, CA), where libraries were prepared with Nextera Tn5 transposase, amplified, assessed for quality control, and sequenced with Illumina High-throughput sequencing, with >60 million total reads of paired-end sequencing at 75–150 bp range. Analysis was performed in R. Briefly, raw sequences were mapped to the human reference genome hg38 (UCSC) using Rsubread (RRID:SCR_016945), followed by sorting and indexing with Rsamtools.^67^^,^^68^ Proper pairs were identified with the GenomicAlignments, with reads normalized per million mapped reads and visualized in the Integrative Genomics Viewer (IGV, Broad Institute, RRID:SCR_011793).^70^ Peak calling was accomplished in R through use of Herper to establish a miniconda environment in R studio and subsequent use of the MACS2 package (RRID:SCR_013291).^69^ Blacklisted regions of the genome were excluded using the DAC exclusion list regionguttrs of the hg38 human reference genome (ENCSR636HFF). Annotation was performed with the ChIPseeker package (RRID:SCR_021322) and UCSC hg38 gene models. DESeq2 (RRID:SCR_015687) was used for differential accessibility analysis between RH30 scramble vs. RH30 MyoD^Δ^ cell samples.^71^^,^^72^ Differentially accessible peaks of genes were filtered for a fold change >25% and a false discovery rate (FDR) of <0.05 with the Benjamini-Hochberg correction. Raw sequences and analyzed files were deposited on GEO (GEO GSE224179).

#### ChIP-seq

Raw sequencing files which captured DNA sequences bound by MyoD in the human FN-RMS cell line, RD, and the FP-RMS cell line, RH4, were retrieved through GEO and analyzed for genes bound by MyoD in RMS (GEO GSE50413, GEO GSE83726).^47^^,^^48^ ChIP-seq analysis was carried out with the same pipeline outlined for ATAC-seq analysis.

#### Bisulfite PCR

In silico investigation of CYLD gene methylation status in RMS cells was performed using Cancer Cell Line Encyclopedia (CCLE) dataset. A total of 155,584 bp of long genomic DNA sequence was downloaded from the NCBI Gene database, starting from Chr 16 at position 50,694,216, with the CYLD gene located between 47,871 and 107,720 bp, containing all highly methylated loci identified in our *in silico* data divided into a total of 8 loci (Table S2). An Ubuntu program was developed with C++ to generate a new genomic DNA sequence by converting all cytosines (C) to thymines (T). This program is also utilized to select primer sets for different loci from the newly created genomic DNA sequence. MyoD in RH30 cells was knocked out using CRISPR technology, using two different sets of sgRNA targeting MyoD gene as MyoD sgRNA G1 + MyoD sgRNA C2 (as Pair 1) and MyoD sgRNA G2 + MyoD sgRNA C3 (as Pair 2), as described above. RH30 cells, either with or without MyoD knockout, were harvested by trypsinization, washed twice with PBS, and pelleted for DNA isolation using the Qiagen DNA Blood Kit (Qiagen, cat# 51104). Bisulfite treatment of the isolated DNA was performed using the Epitect Bisulfite Kit (Qiagen, cat# 59104), following the manufacturer’s protocol. Two micrograms (2 μg) of isolated DNA per sample was diluted in autoclaved DDW to a final volume of 20 μL. Then, 85 μL of bisulfite mix and 35 μL of DNA protection buffer were added to each DNA sample. Bisulfite treatment was carried out using a thermocycler under the following conditions: 95°C (5 min) → 60°C (25 min) → 95°C (5 min) → 60°C (85 min) → 95°C (5 min) → 60°C (175 min) → hold at 20°C. The bisulfite-treated DNA was purified using the spin column provided in the kit and eluted in 40 μL of EB buffer. The eluted DNA was further diluted to 100 μL with DDW, and 2 μL of the bisulfite-treated DNA was used for PCR amplification with Platinum II Taq hot-start DNA polymerase (Thermo Fisher, #14966001 or #11304011). The PCR reaction was set up with.

(1) 1× Platinum II buffer.
(2) 200 μM of each dNTP.
(3) 0.5 pmol/μL of each primer.
(4) 0.4 μL Taq polymerase.
(5) Final reaction volume: 50 μL.

The amplified PCR products were mixed with an additional 50 μL of PCR reaction mixture (without template DNA) and A-tailed for 1 h at 72°C (only #14966001 Taq was used in this step). A-tailed DNA was separated on a 2% agarose gel, and PCR bands were excised and purified using the Qiagen Gel Extraction Kit (#28704). The extracted DNA was then ligated into the pCRII-Topo vector using the TOPO TA Cloning Kit (Thermo Fisher, #K457502), following the manufacturer’s protocol. Briefly, 1 μL of high-salt solution was added to 4 μL of the purified PCR product, followed by the addition 1 μL of the pCRII-Topo vector. The ligation reaction was incubated at room temperature for 5 min. Ligated DNA was transformed into One Shot TOPO10 chemically competent E. coli and plated on LB agar plates containing 20 μg/mL X-GAL and 1 mM IPTG. White colonies were selected, grown in LB broth, and plasmid DNA was purified using the Qiagen Plasmid Mini Prep Kit (#27106). Sanger sequencing was performed on the purified plasmid DNA. The sequencing results were formatted into FASTA format, along with the original genomic DNA sequence downloaded from the NCBI Gene database. Sequence alignment was carried out using Clustal Omega (https://www.ebi.ac.uk/jdispatcher/msa/clustalo).

### Quantification and statistical analysis

All statistical analyses were performed with appropriate statistical tests on GraphPad Prism (Dotmatics, Version 8, RRID:SCR_002798) or in R. Unless otherwise noted, unpaired Student’s t test were used to calculate *p*-values. RNA expression by qPCR analysis was calculated with the ddCT method with *GAPDH* as the internal control. Enrichment of MyoD binding in ChIP assays was calculated with the percent input method. In cell death experiments, percent cell death was normalized to dead cells of control treated cells for all experiments, unless otherwise noted. All data represent the mean ± Standard Error of the Mean. Corresponding appropriate statical tests are shown in footnotes of Figure legends when appropriate.

## Supplemental information

Document S1. Figures S1–S7 and Tables S1 and S2

Table S3. Oligonucleotide sequences, related to Star★Methods

## References

[1] Ries L.A.G., Smith M.A., Gurney J.G., Linet M., Tamra T., Young J.L., Bunin G.R. (1999).

[2] Perez E.A., Kassira N., Cheung M.C., Koniaris L.G., Neville H.L., Sola J.E. (2011). Rhabdomyosarcoma in children: a SEER population based study. J. Surg. Res..

[3] Skapek S.X., Ferrari A., Gupta A.A., Lupo P.J., Butler E., Shipley J., Barr F.G., Hawkins D.S. (2019). Rhabdomyosarcoma. Nat. Rev. Dis. Primers.

[4] Haduong J.H., Heske C.M., Allen-Rhoades W., Xue W., Teot L.A., Rodeberg D.A., Donaldson S.S., Weiss A., Hawkins D.S., Venkatramani R. (2022). An update on rhabdomyosarcoma risk stratification and the rationale for current and future Children's Oncology Group clinical trials. Pediatr. Blood Cancer.

[5] Malempati S., Hawkins D.S. (2012). Rhabdomyosarcoma: review of the Children's Oncology Group (COG) Soft-Tissue Sarcoma Committee experience and rationale for current COG studies. Pediatr. Blood Cancer.

[6] Pappo A.S., Anderson J.R., Crist W.M., Wharam M.D., Breitfeld P.P., Hawkins D., Raney R.B., Womer R.B., Parham D.M., Qualman S.J., Grier H.E. (1999). Survival after relapse in children and adolescents with rhabdomyosarcoma: A report from the Intergroup Rhabdomyosarcoma Study Group. J. Clin. Oncol..

[7] Fletcher C.D.M., Bridge J.A., Hogendoorn P.C.W. (2013).

[8] Agaram N.P. (2022). Evolving classification of rhabdomyosarcoma. Histopathology.

[9] Arnold M.A., Barr F.G. (2017). Molecular diagnostics in the management of rhabdomyosarcoma. Expert Rev. Mol. Diagn..

[10] Shern J.F., Selfe J., Izquierdo E., Patidar R., Chou H.C., Song Y.K., Yohe M.E., Sindiri S., Wei J., Wen X. (2021). Genomic Classification and Clinical Outcome in Rhabdomyosarcoma: A report from an International Consortium. J. Clin. Oncol..

[11] Scrable H., Cavenee W., Ghavimi F., Lovell M., Morgan K., Sapienza C. (1989). A model for embryonal rhabdomyosarcoma tumorigenesis that involves genome imprinting. Proc. Natl. Acad. Sci. USA.

[12] Barr F.G. (2001). Gene fusions involving PAX and FOX family members in alveolar rhabdomyosarcoma. Oncogene.

[13] Hibbitts E., Chi Y.Y., Hawkins D.S., Barr F.G., Bradley J.A., Dasgupta R., Meyer W.H., Rodeberg D.A., Rudzinski E.R., Spunt S.L. (2019). Refinement of risk stratification for childhood rhabdomyosarcoma using FOXO1 fusion status in addition to established clinical outcome predictors: A report from the Children's Oncology Group. Cancer Med..

[14] Skapek S.X., Anderson J., Barr F.G., Bridge J.A., Gastier-Foster J.M., Parham D.M., Rudzinski E.R., Triche T., Hawkins D.S. (2013). PAX-FOXO1 fusion status drives unfavorable outcome for children with rhabdomyosarcoma: a children's oncology group report. Pediatr. Blood Cancer.

[15] Kashi V.P., Hatley M.E., Galindo R.L. (2015). Probing for a deeper understanding of rhabdomyosarcoma: insights from complementary model systems. Nat. Rev. Cancer.

[16] Dias P., Chen B., Dilday B., Palmer H., Hosoi H., Singh S., Wu C., Li X., Thompson J., Parham D. (2000). Strong immunostaining for myogenin in rhabdomyosarcoma is significantly associated with tumors of the alveolar subclass. Am. J. Pathol..

[17] Sebire N.J., Malone M. (2003). Myogenin and MyoD1 expression in paediatric rhabdomyosarcomas. J. Clin. Pathol..

[18] Tapscott S.J. (2005). The circuitry of a master switch: Myod and the regulation of skeletal muscle gene transcription. Development.

[19] Weintraub H., Davis R., Tapscott S., Thayer M., Krause M., Benezra R., Blackwell T.K., Turner D., Rupp R., Hollenberg S. (1991). The myoD gene family: nodal point during specification of the muscle cell lineage. Science.

[20] Buckingham M., Rigby P.W.J. (2014). Gene regulatory networks and transcriptional mechanisms that control myogenesis. Dev. Cell.

[21] Chargé S.B.P., Rudnicki M.A. (2004). Cellular and molecular regulation of muscle regeneration. Physiol. Rev..

[22] Keller C., Guttridge D.C. (2013). Mechanisms of impaired differentiation in rhabdomyosarcoma. FEBS J..

[23] Saab R., Spunt S.L., Skapek S.X. (2011). Myogenesis and rhabdomyosarcoma the Jekyll and Hyde of skeletal muscle. Curr. Top. Dev. Biol..

[24] Patel A.G., Chen X., Huang X., Clay M.R., Komorova N., Krasin M.J., Pappo A., Tillman H., Orr B.A., McEvoy J. (2022). The myogenesis program drives clonal selection and drug resistance in rhabdomyosarcoma. Dev. Cell.

[25] Stewart E., McEvoy J., Wang H., Chen X., Honnell V., Ocarz M., Gordon B., Dapper J., Blankenship K., Yang Y. (2018). Identification of therapeutic targets in rhabdomyosarcoma through integrated genomic, epigenomic, and proteomic analyses. Cancer Cell.

[26] Bakkar N., Guttridge D.C. (2010). NF-kappaB signaling: a tale of two pathways in skeletal myogenesis. Physiol. Rev..

[27] Wang H., Garzon R., Sun H., Ladner K.J., Singh R., Dahlman J., Cheng A., Hall B.M., Qualman S.J., Chandler D.S. (2008). NF-kappaB-YY1-miR-29 regulatory circuitry in skeletal myogenesis and rhabdomyosarcoma. Cancer Cell.

[28] Beg A.A., Baltimore D. (1996). An essential role for NF-kappaB in preventing TNF-alpha-induced cell death. Science.

[29] Chu Z.L., McKinsey T.A., Liu L., Gentry J.J., Malim M.H., Ballard D.W. (1997). Suppression of tumor necrosis factor-induced cell death by inhibitor of apoptosis c-IAP2 is under NF-kappaB control. Proc. Natl. Acad. Sci. USA.

[30] Liu Z.G., Hsu H., Goeddel D.V., Karin M. (1996). Dissection of TNF receptor 1 effector functions: JNK activation is not linked to apoptosis while NF-kappaB activation prevents cell death. Cell.

[31] Orlowski R.Z., Baldwin A.S. (2002). NF-kappaB as a therapeutic target in cancer. Trends Mol. Med..

[32] Wang C.Y., Cusack J.C., Liu R., Baldwin A.S. (1999). Control of inducible chemoresistance: enhanced anti-tumor therapy through increased apoptosis by inhibition of NF-kappaB. Nat. Med..

[33] Wang C.Y., Mayo M.W., Baldwin A.S. (1996). TNF- and cancer therapy-induced apoptosis: potentiation by inhibition of NF-kappaB. Science.

[34] Balkhi M.Y., Iwenofu O.H., Bakkar N., Ladner K.J., Chandler D.S., Houghton P.J., London C.A., Kraybill W., Perrotti D., Croce C.M. (2013). miR-29 acts as a decoy in sarcomas to protect the tumor suppressor A20 mRNA from degradation by HuR. Sci. Signal..

[35] Brockman J.A., Scherer D.C., McKinsey T.A., Hall S.M., Qi X., Lee W.Y., Ballard D.W. (1995). Coupling of a signal response domain in I kappa B alpha to multiple pathways for NF-kappa B activation. Mol. Cell Biol..

[36] Baldwin A.S. (2001). Control of oncogenesis and cancer therapy resistance by the transcription factor NF-kappaB. J. Clin. Investig..

[37] Missiaglia E., Williamson D., Chisholm J., Wirapati P., Pierron G., Petel F., Concordet J.P., Thway K., Oberlin O., Pritchard-Jones K. (2012). PAX3/FOXO1 fusion gene status is the key prognostic molecular marker in rhabdomyosarcoma and significantly improves current risk stratification. J. Clin. Oncol..

[38] Williamson D., Missiaglia E., de Reyniès A., Pierron G., Thuille B., Palenzuela G., Thway K., Orbach D., Laé M., Fréneaux P. (2010). Fusion gene-negative alveolar rhabdomyosarcoma is clinically and molecularly indistinguishable from embryonal rhabdomyosarcoma. J. Clin. Oncol..

[39] Sartorelli V., Puri P.L. (2018). Shaping gene expression by landscaping chromatin architecture: lessons from a master. Mol. Cell.

[40] Van Antwerp M.E., Chen D.G., Chang C., Prochownik E.V. (1992). A point mutation in the MyoD basic domain imparts c-Myc-like properties. Proc. Natl. Acad. Sci. USA.

[41] Dempster J.M., Boyle I., Vazquez F., Root D.E., Boehm J.S., Hahn W.C., Tsherniak A., McFarland J.M. (2021). Chronos: a cell population dynamics model of CRISPR experiments that improves inference of gene fitness effects. Genome Biol..

[42] Dempster J.M., Rossen J., Kazachkova M., Pan J., Kugener G., Root D.E., Tsherniak A. (2019). Extracting biological insights from the project achilles genome-scale CRISPR screens in cancer cell lines. bioRxiv.

[43] Takahashi K., Yamanaka S. (2006). Induction of pluripotent stem cells from mouse embryonic and adult fibroblast cultures by defined factors. Cell.

[44] Hanahan D., Weinberg R.A. (2011). Hallmarks of cancer: the next generation. Cell.

[45] Wang D.J., Ratnam N.M., Byrd J.C., Guttridge D.C. (2014). NF-kappaB functions in tumor initiation by suppressing the surveillance of both innate and adaptive immune cells. Cell Rep..

[46] Dunham I., Kundaje A., Aldred S.F., Collins P.J., Davis C.A., Doyle F., Epstein C.B., Frietze S., Harrow J., Kaul R. (2012). An integrated encyclopedia of DNA elements in the human genome. Nature.

[47] Gryder B.E., Yohe M.E., Chou H.C., Zhang X., Marques J., Wachtel M., Schaefer B., Sen N., Song Y., Gualtieri A. (2017). PAX3-FOXO1 Establishes myogenic super enhancers and confers BET bromodomain vulnerability. Cancer Discov..

[48] MacQuarrie K.L., Yao Z., Fong A.P., Diede S.J., Rudzinski E.R., Hawkins D.S., Tapscott S.J. (2013). Comparison of genome-wide binding of MyoD in normal human myogenic cells and rhabdomyosarcomas identifies regional and local suppression of promyogenic transcription factors. Mol. Cell Biol..

[49] Gardiner-Garden M., Frommer M. (1987). CpG islands in vertebrate genomes. J. Mol. Biol..

[50] Kent W.J., Sugnet C.W., Furey T.S., Roskin K.M., Pringle T.H., Zahler A.M., Haussler D. (2002). The human genome browser at UCSC. Genome Res..

[51] Shintaku J., Peterson J.M., Talbert E.E., Gu J.M., Ladner K.J., Williams D.R., Mousavi K., Wang R., Sartorelli V., Guttridge D.C. (2016). MyoD regulates skeletal muscle oxidative metabolism cooperatively with alternative NF-kappaB. Cell Rep..

[52] Lyko F. (2018). The DNA methyltransferase family: a versatile toolkit for epigenetic regulation. Nat. Rev. Genet..

[53] Koga Y., Pelizzola M., Cheng E., Krauthammer M., Sznol M., Ariyan S., Narayan D., Molinaro A.M., Halaban R., Weissman S.M. (2009). Genome-wide screen of promoter methylation identifies novel markers in melanoma. Genome Res..

[54] Lork M., Verhelst K., Beyaert R. (2017). CYLD, A20 and OTULIN deubiquitinases in NF-kappaB signaling and cell death: so similar, yet so different. Cell Death Differ..

[55] Bignell G.R., Warren W., Seal S., Takahashi M., Rapley E., Barfoot R., Green H., Brown C., Biggs P.J., Lakhani S.R. (2000). Identification of the familial cylindromatosis tumour-suppressor gene. Nat. Genet..

[56] Massoumi R. (2011). CYLD: a deubiquitination enzyme with multiple roles in cancer. Future Oncol..

[57] Zhang Q., Lenardo M.J., Baltimore D. (2017). 30 years of NF-kappaB: a blossoming of relevance to human pathobiology. Cell.

[58] Cleary M.M., Mansoor A., Settelmeyer T., Ijiri Y., Ladner K.J., Svalina M.N., Rubin B.P., Guttridge D.C., Keller C. (2017). NFκB signaling in alveolar rhabdomyosarcoma. Dis. Model. Mech..

[59] Knapp J.R., Davie J.K., Myer A., Meadows E., Olson E.N., Klein W.H. (2006). Loss of myogenin in postnatal life leads to normal skeletal muscle but reduced body size. Development.

[60] Megeney L.A., Kablar B., Garrett K., Anderson J.E., Rudnicki M.A. (1996). MyoD is required for myogenic stem cell function in adult skeletal muscle. Genes Dev..

[61] Dharia N.V., Kugener G., Guenther L.M., Malone C.F., Durbin A.D., Hong A.L., Howard T.P., Bandopadhayay P., Wechsler C.S., Fung I. (2021). A first-generation pediatric cancer dependency map. Nat. Genet..

[62] Hsu J.Y., Danis E.P., Nance S., O'Brien J.H., Gustafson A.L., Wessells V.M., Goodspeed A.E., Talbot J.C., Amacher S.L., Jedlicka P. (2022). SIX1 reprograms myogenic transcription factors to maintain the rhabdomyosarcoma undifferentiated state. Cell Rep..

[63] Bergstrom D.A., Penn B.H., Strand A., Perry R.L.S., Rudnicki M.A., Tapscott S.J. (2002). Promoter-specific regulation of MyoD binding and signal transduction cooperate to pattern gene expression. Mol. Cell.

[64] Tenente I.M., Hayes M.N., Ignatius M.S., McCarthy K., Yohe M., Sindiri S., Gryder B., Oliveira M.L., Ramakrishnan A., Tang Q. (2017). Myogenic regulatory transcription factors regulate growth in rhabdomyosarcoma. eLife.

[65] Guttridge D.C., Albanese C., Reuther J.Y., Pestell R.G., Baldwin A.S. (1999). NF-kappaB controls cell growth and differentiation through transcriptional regulation of cyclin D1. Mol. Cell Biol..

[66] Guttridge D.C., Mayo M.W., Madrid L.V., Wang C.Y., Baldwin A.S. (2000). NF-kappaB-induced loss of MyoD messenger RNA: possible role in muscle decay and cachexia. Science.

[67] Liao Y., Smyth G.K., Shi W. (2019). The R package Rsubread is easier, faster, cheaper and better for alignment and quantification of RNA sequencing reads. Nucleic Acids Res..

[68] Li H., Handsaker B., Wysoker A., Fennell T., Ruan J., Homer N., Marth G., Abecasis G., Durbin R., 1000 Genome Project Data Processing Subgroup (2009). The sequence alignment/map format and SAMtools. Bioinformatics.

[69] Zhang Y., Liu T., Meyer C.A., Eeckhoute J., Johnson D.S., Bernstein B.E., Nusbaum C., Myers R.M., Brown M., Li W., Liu X.S. (2008). Model-based analysis of ChIP-Seq (MACS). Genome Biol..

[70] Lawrence M., Huber W., Pagès H., Aboyoun P., Carlson M., Gentleman R., Morgan M.T., Carey V.J. (2013). Software for computing and annotating genomic ranges. PLoS Comput. Biol..

[71] Yu G., Wang L.G., He Q.Y. (2015). ChIPseeker: an R/Bioconductor package for ChIP peak annotation, comparison and visualization. Bioinformatics.

[72] Love M.I., Huber W., Anders S. (2014). Moderated estimation of fold change and dispersion for RNA-seq data with DESeq2. Genome Biol..

[73] Carvalho B.S., Irizarry R.A. (2010). A framework for oligonucleotide microarray preprocessing. Bioinformatics.

[74] Smyth G.K., Gentleman R., Carey V.J., Huber W., Irizarry R.A., Dudoit S. (2005). Bioinformatics and Computational Biology Solutions Using R and Bioconductor.

[75] Wang H., Hertlein E., Bakkar N., Sun H., Acharyya S., Wang J., Carathers M., Davuluri R., Guttridge D.C. (2007). NF-kappaB regulation of YY1 inhibits skeletal myogenesis through transcriptional silencing of myofibrillar genes. Mol. Cell Biol..

[76] Ratnam N.M., Peterson J.M., Talbert E.E., Ladner K.J., Rajasekera P.V., Schmidt C.R., Dillhoff M.E., Swanson B.J., Haverick E., Kladney R.D. (2017). NF-kappaB regulates GDF-15 to suppress macrophage surveillance during early tumor development. J. Clin. Investig..

