# Supplementary material for: MyoD is essential in rhabdomyosarcoma by promoting survival through differentiation and CYLD
Source: iScience. 2025 Jul 18;28(8):113149. doi: 10.1016/j.isci.2025.113149 (PMC12341531; doi:10.1016/j.isci.2025.113149)
Supplement: Document S1. Figures S1–S7 and Tables S1 and S2 [file mmc1.pdf]

iScience, Volume *28*

Supplemental information

MyoD is essential in rhabdomyosarcoma by promoting survival through differentiation and CYLD

Alexander R. Oles, Peter Y. Yu, Abasi-ama Udeme, Sudarshana Sharma, Priya Londhe, Benjamin R. Pryce, Erin E. Talbert, Eric M. Hill, Carlos J. Miranda, Brian K. Kaspar, Michael A. Arnold, Jack Hyland, Cheryl A. London, Peter J. Houghton, David J. Wang, Ryan D. Roberts, and Denis C. Guttridge

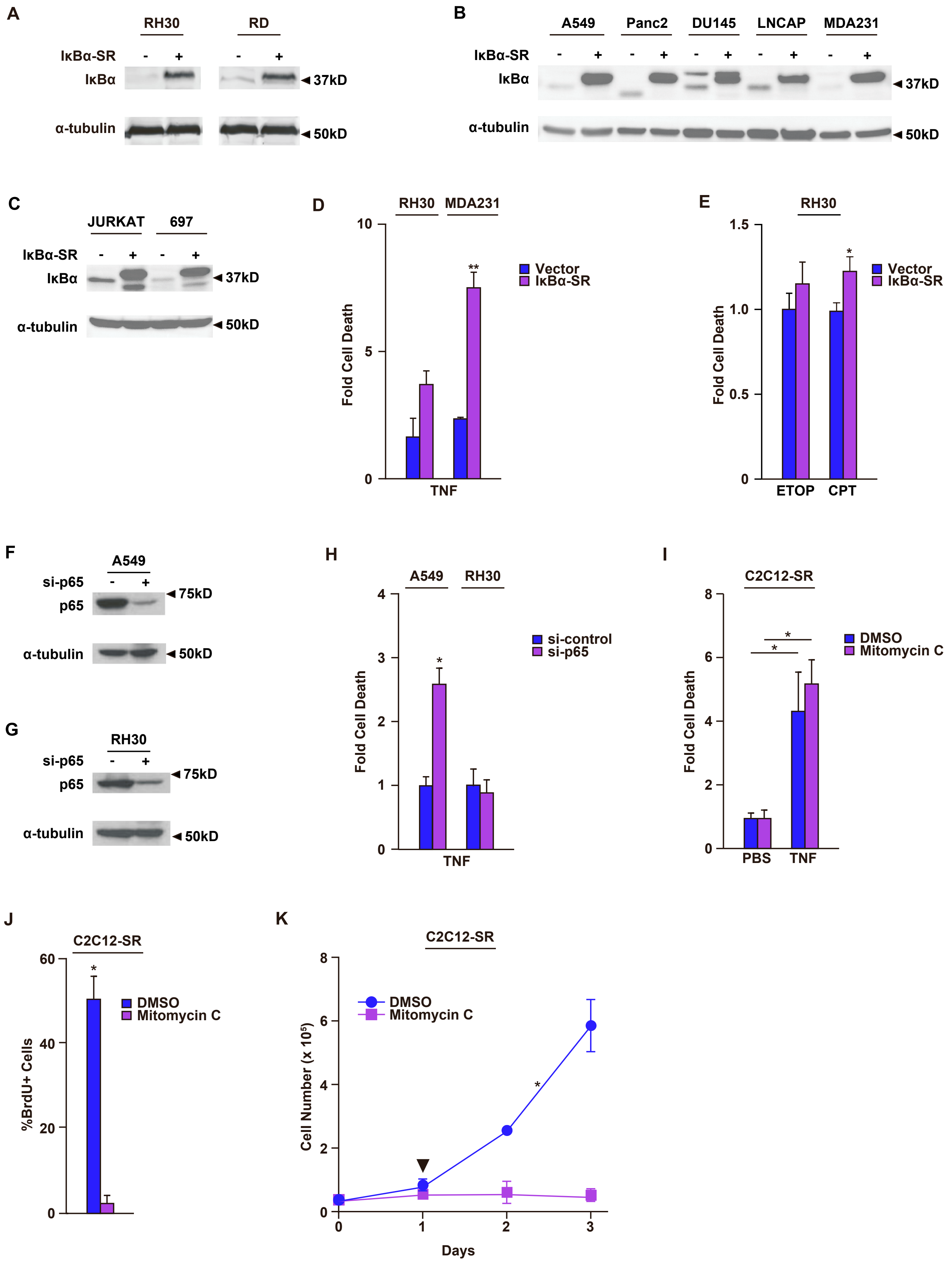
A
RH30
RD
IκBα-SR
IκBα
α-tubulin
37kD
50kD
B
A549
Panc2
DU145
LNCAP
MDA231
IκBα-SR
IκBα
α-tubulin
37kD
50kD
C
JURKAT
697
IκBα-SR
IκBα
α-tubulin
37kD
50kD
D
RH30
MDA231
Fold Cell Death
Vector
IκBα-SR
TNF
E
RH30
Fold Cell Death
Vector
IκBα-SR
ETOP
CPT
F
A549
si-p65
p65
α-tubulin
75kD
50kD
G
RH30
si-p65
p65
α-tubulin
75kD
50kD
H
A549
RH30
Fold Cell Death
si-control
si-p65
TNF
I
C2C12-SR
Fold Cell Death
DMSO
Mitomycin C
PBS
TNF
J
C2C12-SR
%BrdU+ Cells
DMSO
Mitomycin C
K
C2C12-SR
Cell Number (x 10⁵)
DMSO
Mitomycin C
Days

Figure S1. RMS resistance to cellular stress and C2C12 myotubes resistance to stress conferred by myogenic differentiation are independent NF-κB activity, related to Figure 1. (A) Immunoblot of IκBα representing expression of the IκBα super repressor (IκBα-SR) in FP (RH30) and FN (RD) cells infected with retrovirus expressing IκBα-SR (+) and Vector-control (-), with α–tubulin used as a loading control. **(B, C)** Immunoblot of IκBα in epithelial (A549, Panc2, DU145, LNCAP, MDA231) and hematological (Jurkat, 697) cancer cell lines infected with IκBα-SR (+) or Vector-control (-) viruses, with α–tubulin used as a loading control. **(D)** RH30 and MDA231 cells expressing IκBα-SR were treated with TNF (10 ng/mL) for 24 hrs and cell death was subsequently measured by Annexin V and flow cytometry, n = 3. **(E)** RH30-SR cells were treated with etoposide (ETOP) or camptothecin (CTP) (10 μM) for 24 hrs and cell death was measured as in **(D)** normalized to treated RH30-vector infected control cells, n = 3. **(F, G)** Western blots of p65 in A549 and in RH30 cells following transfection with siRNA targeting p65 or scrambled control with α–tubulin expression used as a loading control. **(H)** Transfected cells in **(F)** or **(G)** were treated with TNF (5 ng/mL) for 24 hrs and cell death was subsequently measured, n = 3. **(I)** C2C12 myoblasts expressing IκBα-SR were treated with mitomycin C (20 μg/mL) or DMSO for 3 hrs, followed by PBS or TNF for 24 hrs. Cell death was subsequently measured, normalized to death in PBS treated cells for each condition, and analyzed by an unpaired Student's t test, n = 3. **(J)** BrdU (10 μM) positivity was assessed in myoblasts expressing IκBα-SR following treatment with DMSO or mitomycin C (20 μg/mL) via flow cytometry, n = 3. **(K)** Growth curve of C2C12 myoblasts expressing IκBα-SR following treatment with DMSO or mitomycin C (20 μg/mL), n = 3. Data with error bars are depicted as mean ± SEM, * $p < 0.05$; ** $p < 0.01$.

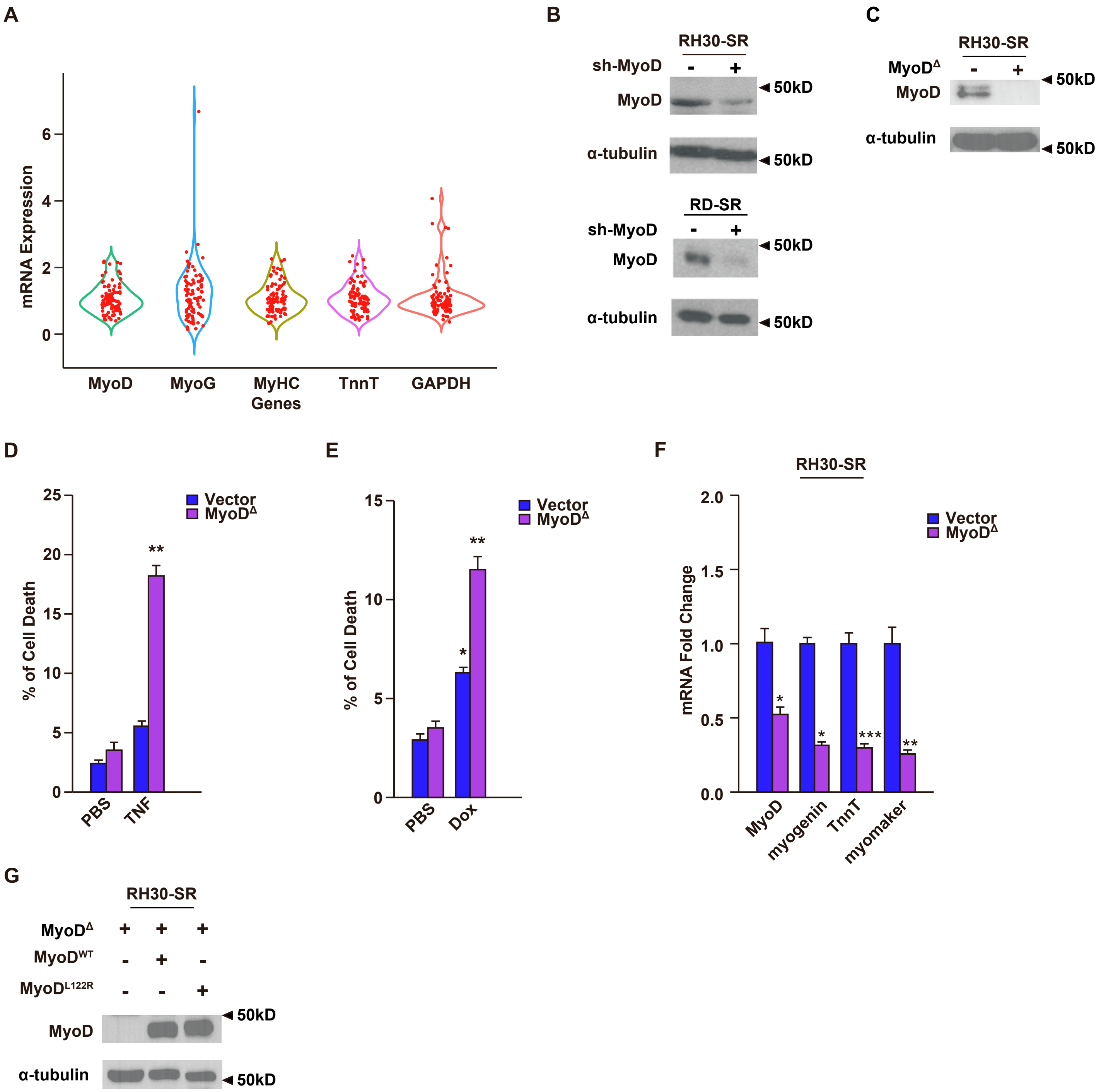

Figure S2. Loss of MyoD decreases the RMS differentiation phenotype and promotes stress-induced cell death, related to Figure 2. (A) Violin plot representative of single cell qPCR expression of MyoD, myogenin, TnnT, and MyHC from RH30 cells. GAPDH was used as an internal control. **(B)** Western blots of MyoD in RH30-SR and RD-SR cells following expression of a scrambled-control sh-RNA (-) or an sh-RNA targeting MyoD (+). **(C)** Same condition as in **(A)** following CRISPR/Cas9 deletion of MyoD ($MyoD^{\Delta}$). **(D, E)** Absolute cell death of RH30-SR Vector or $MyoD^{\Delta}$ cells treated with PBS control, TNF **(D)**, or DOX **(E)** for 24 hrs. **(F)** qPCR analysis comparing expression of MyoD and its respective target genes in RH30-SR $MyoD^{\Delta}$ cells, n = 3. **(G)** Western blots of MyoD in RH30-SR $MyoD^{\Delta}$ cells reconstituted with either wildtype MyoD or the MyoD mutant, $MyoD^{L122R}$. α-tubulin used as a loading control. Data with error bars are depicted as mean ± SEM, * $p < 0.05$; ** $p < 0.01$; *** $p < 0.001$.

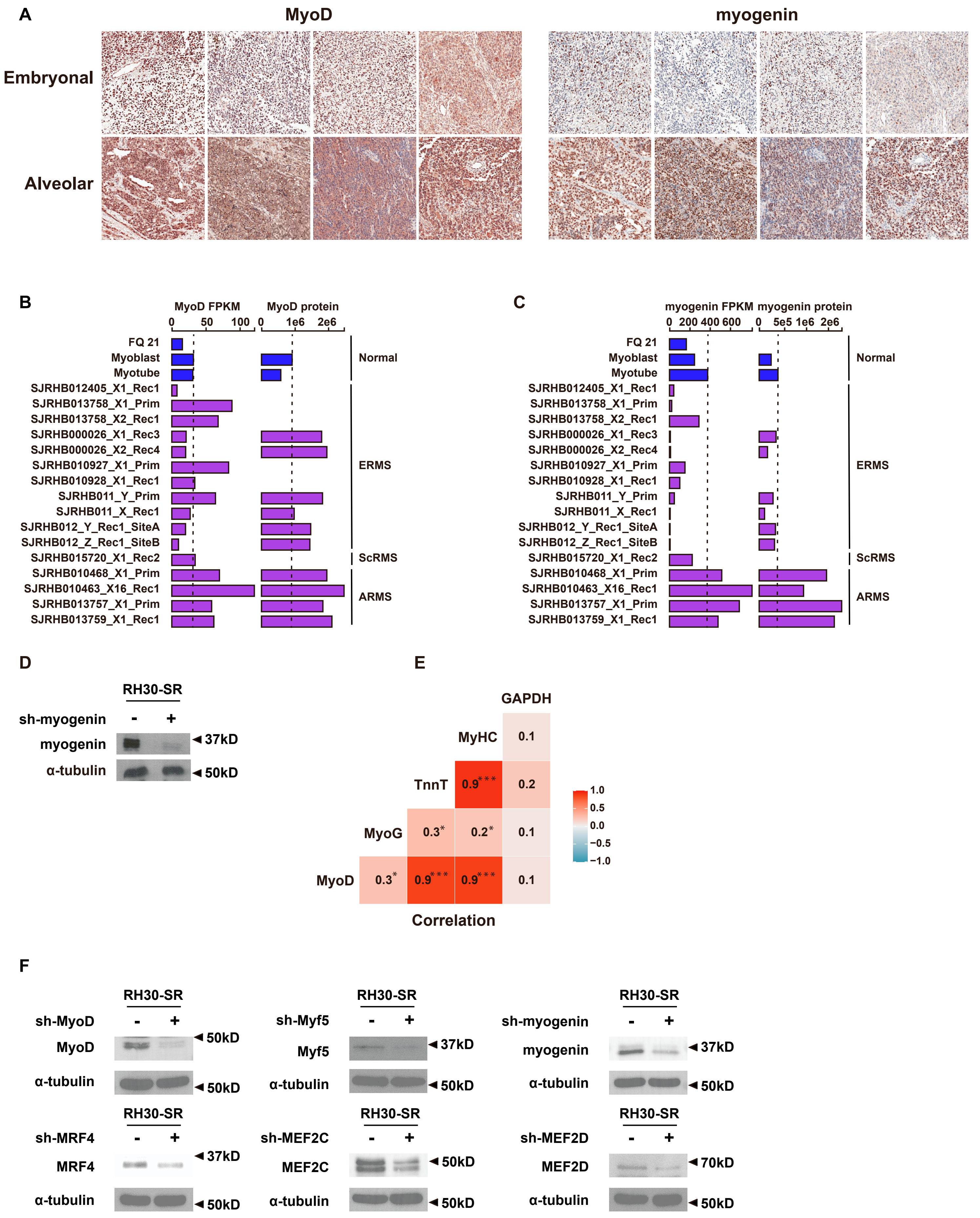

A
MyoD
myogenin
Embryonal
Alveolar
B
MyoD FPKM
MyoD protein
0 50 100
0 1e6 2e6
FQ 21
Myoblast
Myotube
SJRHB012405_X1_Rec1
SJRHB013758_X1_Prim
SJRHB013758_X2_Rec1
SJRHB000026_X1_Rec3
SJRHB000026_X2_Rec4
SJRHB010927_X1_Prim
SJRHB010928_X1_Rec1
SJRHB011_Y_Prim
SJRHB011_X_Rec1
SJRHB012_Y_Rec1_SiteA
SJRHB012_Z_Rec1_SiteB
SJRHB015720_X1_Rec2
SJRHB010468_X1_Prim
SJRHB010463_X16_Rec1
SJRHB013757_X1_Prim
SJRHB013759_X1_Rec1
Normal
ERMS
ScRMS
ARMS
C
myogenin FPKM
myogenin protein
0 200 400 600
0 5e5 1e6 2e6
D
RH30-SR
sh-myogenin - +
myogenin
α-tubulin
37kD
50kD
E
GAPDH
MyHC 0.1
TnnT 0.9*** 0.2
MyoG 0.3* 0.2* 0.1
MyoD 0.3* 0.9*** 0.9*** 0.1
1.0
0.5
0.0
−0.5
−1.0
Correlation
F
RH30-SR
sh-MyoD - +
MyoD
α-tubulin
50kD
50kD
sh-Myf5 - +
Myf5
37kD
sh-myogenin - +
myogenin
37kD
sh-MRF4 - +
MRF4
37kD
sh-MEF2C - +
MEF2C
50kD
sh-MEF2D - +
MEF2D
70kD

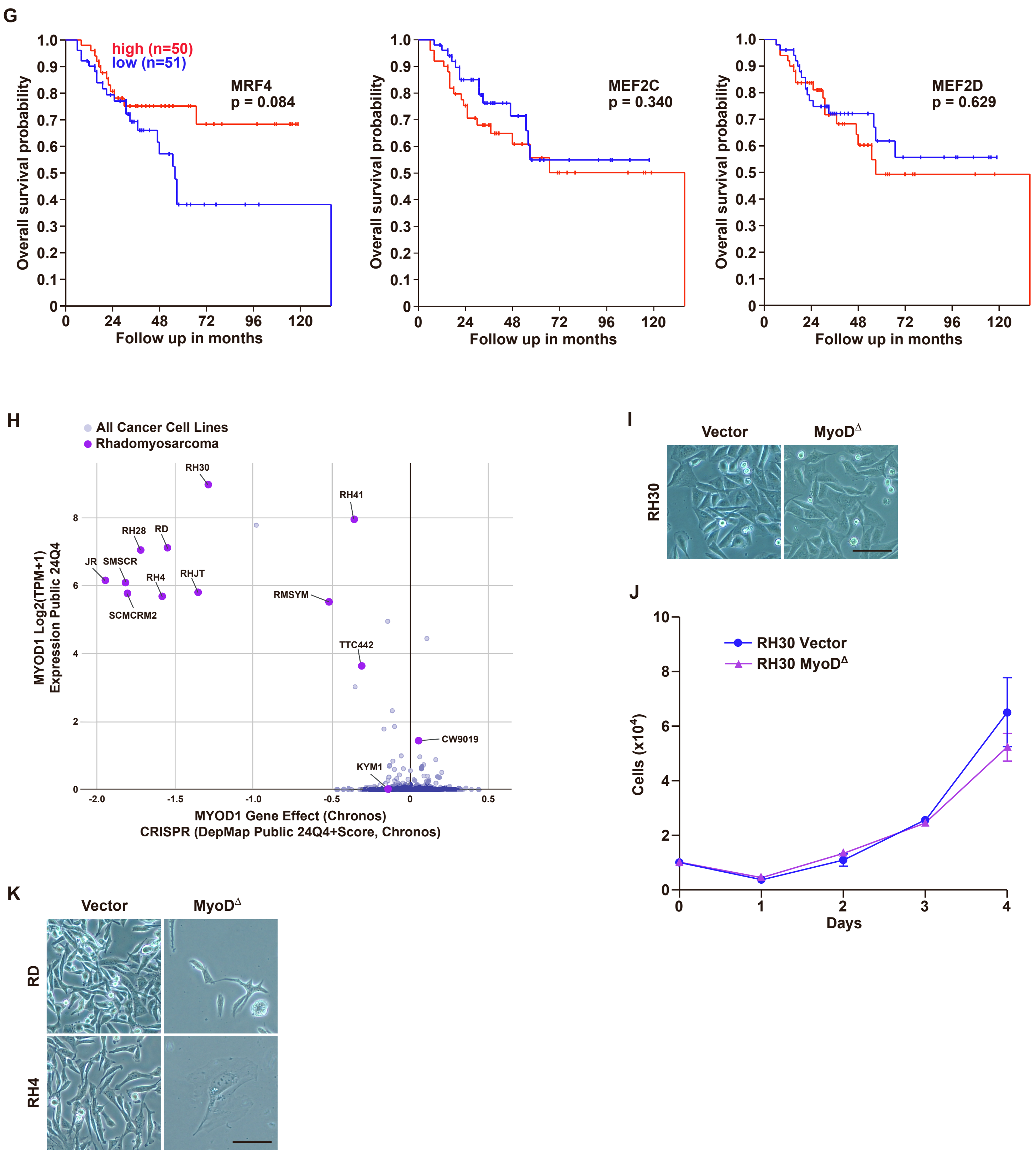
G
high (n=50)
low (n=51)
MRF4
p = 0.084
Overall survival probability
Follow up in months
MEF2C
p = 0.340
Overall survival probability
Follow up in months
MEF2D
p = 0.629
Overall survival probability
Follow up in months
H
All Cancer Cell Lines
Rhadomyosarcoma
RH30
RH41
RH28
RD
JR
SMSCR
RH4
RHJT
SCMCRM2
RMSYM
TTC442
CW9019
KYM1
MYOD1 Log2(TPM+1)
Expression Public 24Q4
MYOD1 Gene Effect (Chronos)
CRISPR (DepMap Public 24Q4+Score, Chronos)
I
Vector
MyoD$^{\Delta}$
RH30
J
RH30 Vector
RH30 MyoD$^{\Delta}$
Cells (x10^{4})
Days
K
Vector
MyoD$^{\Delta}$
RD
RH4

Figure S3. MyoD, but not other myogenic regulator factors, drives RMS survival and correlates with poor patient prognosis, related to Figure 3. (A) Embryonal and alveolar RMS patient tumor sections were stained by immunohistochemistry for MyoD and myogenin; n = 5. **(B, C)** MyoD and myogenin RNA and protein expression in alveolar and embryonal O-PDXs compared to myoblast and myotube cultures from the St. Jude Epigenetic Landscape of Rhabdomyosarcoma Subtypes Dataset. Dotted line denotes the maximum expression of MyoD **(B)** and myogenin **(C)** from cultured muscle cells. **(D)** Western blot of myogenin in RH30-SR cells following expression of a scrambled-control sh-RNA (-) or sh-RNA targeting myogenin (+). α–tubulin was used as a loading control. **(E)** Single cell analysis of correlation of myogenic gene expression determined from RH30 cells. **(F)** Western blot analyses from RH30-SR cells transduced with their respective targeting sh-RNA corresponding to MyoD, myogenin, Myf5, MRF4, MEF2C, and MEF2D (+) compared to scrambled control (-), using α–tubulin as a loading control. **(G)** Kaplan-Meier curve, log-rank test, showing the correlation of both MRF4, MEF2C, and MEF2D expression stratified by median patient expression to overall survival of 101 RMS patients from microarray expression data set.[38] **(H)** CHRONOS Score and log2 expression levels for MyoD in RMS cell lines from DepMap. **(I)** Phase contrast microscopy images of cultured RH30 cells infected with scramble sgRNA (Vector) or sgRNA for MyoD (MyoD$^{\Delta}$); scale bar represents 100 µm. **(J)** Growth curve assays comparing RH30 MyoD$^{\Delta}$ to RH30 Vector control cells, n = 3. **(K)** Phase contrast microscopy images of cultured RD and RH4 RMS cells infected as in **(I)**; scale bar represents 100 µm, * $p < 0.05$, *** $p < 0.001$.

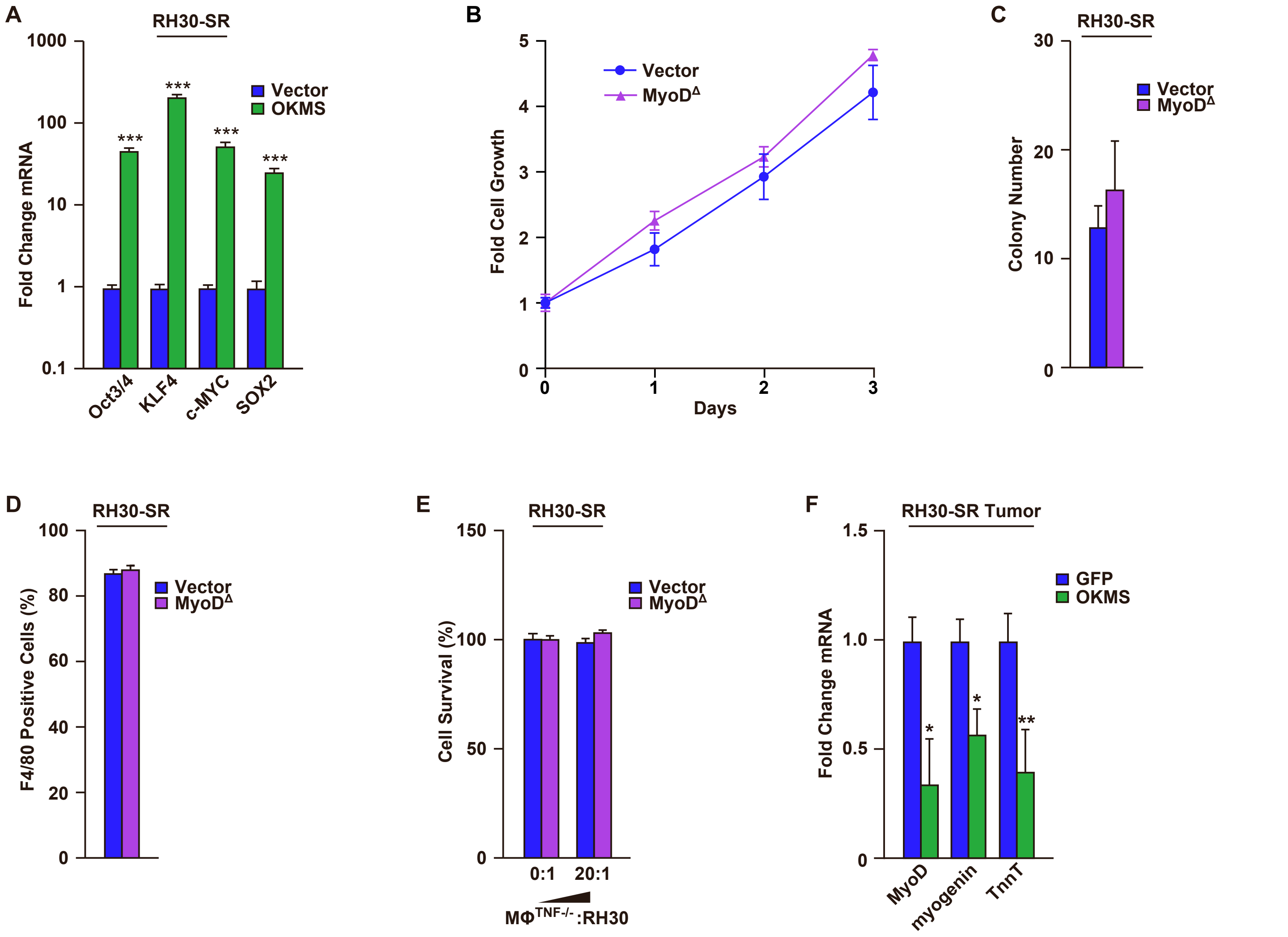

Figure S4. RMS tumors are sensitized to stress-induced death by dedifferentiation through reprogramming factors, related to Figure 4. (A) RH30-SR cells were infected with Vector-control or Yamanaka factors (OKMS) and expression of OKMS factors was analyzed by qPCR, n = 3. **(B)** Growth curve assays comparing RH30-SR MyoD$^{\Delta}$ to RH30-SR Vector control cells, n = 3. **(C)** Cells from **(B)** were utilized for soft agar colony forming assays and total colony number formation was subsequently measured, n = 3. **(D)** RH30-SR Vector and RH30-SR MyoD$^{\Delta}$ cells were injected into the peritoneum of SCID mice and the percentage of F4/80^{+} cells in abdominal fluid was subsequently determined by flow cytometry, n = 3. **(E)** RH30-SR-Vector and RH30-SR-MyoD$^{\Delta}$ cells were co-cultured with activated *TNF*$^{-/-}$ macrophages (0:1 and 20:1) and cell survival was subsequently scored by trypan blue exclusion and compared to baseline cell death, n = 3. **(F)** Xenograft RH30-SR tumors in SCID mice were subsequently injected with lentiviruses expressing Vector control or OKMS factors. After tumors were removed, qPCR was performed to probe for expression of myogenic markers MyoD, myogenin, and TnnT in OKMS compared to GFP control, n = 3. Data with error bars are depicted as mean ± SEM, * $p < 0.05$; ** $p < 0.01$; *** $p < 0.001$.

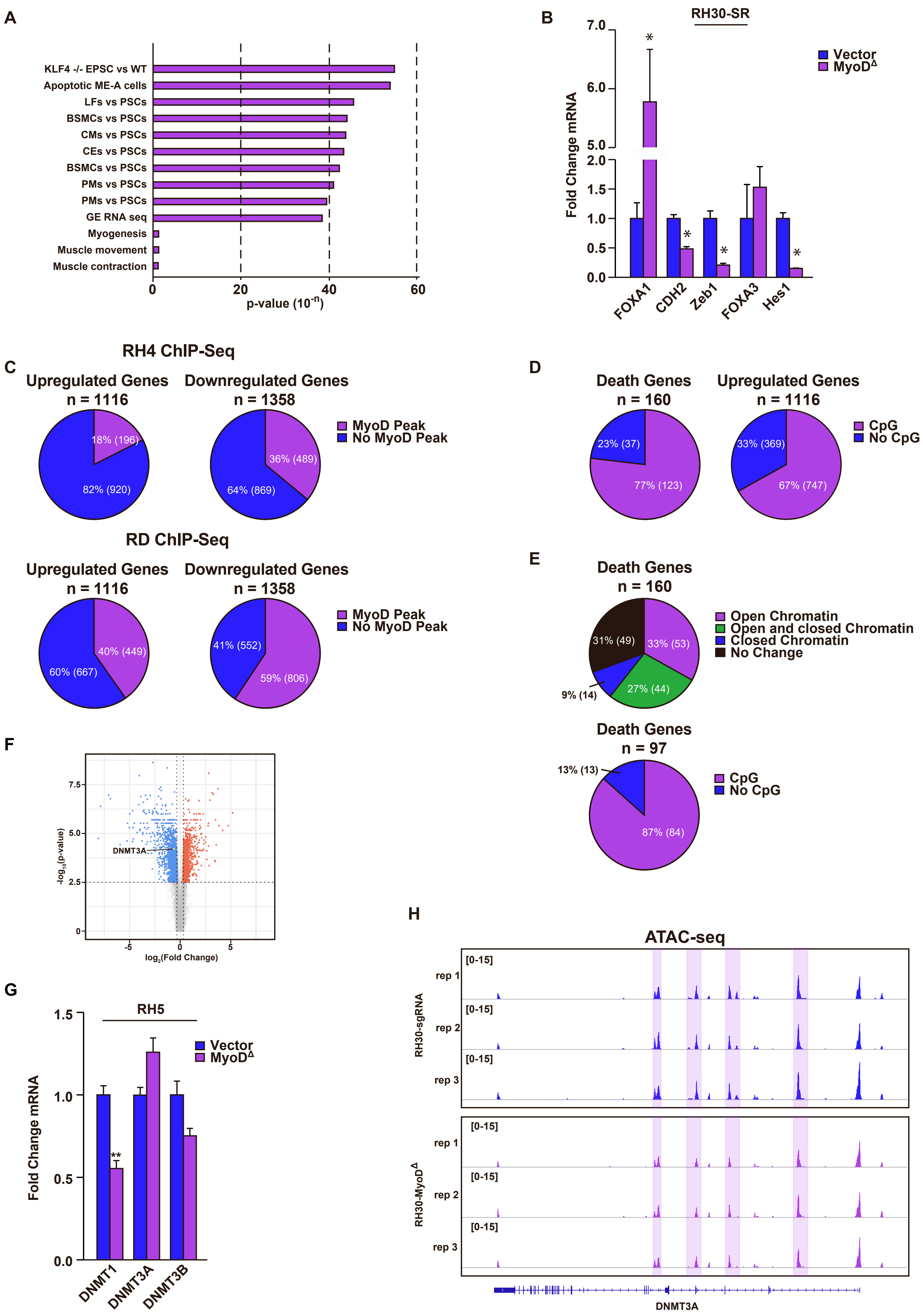
A
KLF4 -/- EPSC vs WT
Apoptotic ME-A cells
LFs vs PSCs
BSMCs vs PSCs
CMs vs PSCs
CEs vs PSCs
BSMCs vs PSCs
PMs vs PSCs
PMs vs PSCs
GE RNA seq
Myogenesis
Muscle movement
Muscle contraction
0
20
40
60
p-value (10^-n)
B
RH30-SR
Vector
MyoD^Δ
Fold Change mRNA
7.0
6.0
5.0
2.0
1.5
1.0
0.5
0.0
*
FOXA1
CDH2
Zeb1
FOXA3
Hes1
RH4 ChIP-Seq
C
Upregulated Genes
n = 1116
18% (196)
82% (920)
Downregulated Genes
n = 1358
36% (489)
64% (869)
MyoD Peak
No MyoD Peak
RD ChIP-Seq
Upregulated Genes
n = 1116
40% (449)
60% (667)
Downregulated Genes
n = 1358
41% (552)
59% (806)
MyoD Peak
No MyoD Peak
D
Death Genes
n = 160
23% (37)
77% (123)
Upregulated Genes
n = 1116
33% (369)
67% (747)
CpG
No CpG
E
Death Genes
n = 160
31% (49)
33% (53)
27% (44)
9% (14)
Open Chromatin
Open and closed Chromatin
Closed Chromatin
No Change
Death Genes
n = 97
13% (13)
87% (84)
CpG
No CpG
F
7.5
5.0
2.5
0
-log10(p-value)
DNMT3A
-5
0
5
log2(Fold Change)
G
RH5
1.5
1.0
0.5
0.0
Fold Change mRNA
Vector
MyoD^Δ
**
DNMT1
DNMT3A
DNMT3B
H
ATAC-seq
RH30-sgRNA
rep 1
rep 2
rep 3
[0-15]
RH30-MyoD^Δ
rep 1
rep 2
rep 3
[0-15]
DNMT3A

Figure S5. MyoD ChIP-seq peaks in RMS cells are associated with death genes and loss of MyoD is associated with more permissive chromatin related to death and DNMT genes, related to Figure 5. (A) Co-expression Atlas pathways enriched for MyoD ChIP-seq binding peaks in RD RMS cells revealed genes associated with pluripotent stem cells (PSCs) and development. **(B)** Stem cell genes identified from **(A)** were measured by qPCR from RH30-SR Vector and RH30-SR MyoD$^{\Delta}$ cells. **(C)** Graphs from combined analyses of RH30-SR MyoD$^{\Delta}$ transcriptomic data and ChIP-seq data reveal percentage of upregulated (n = 1116) and downregulated (n =1358) genes that contain (purple) or lack (blue) MyoD enrichment peaks. **(D)** Graph from *in silico* analysis of CpG islands within the death genes (n = 160) identified from GO enrichment pathways of upregulated genes (n = 1116) and all upregulated genes identified by transcriptomic analysis (Chi-square = 5.92, p = 0.015) **(E)** Representative upper graph denotes ATAC-seq results showing the change in chromatin status following the ablation of MyoD for the 160 death genes identified from GO enrichment pathways, denoted as open (purple), open and closed (green), only closed (blue), or no change (black). Lower graph represents results from *in silico* analysis of CpG islands from genes in the upper graph containing open chromatin regions (n = 97). **(F)** Volcano plot of transcriptomic analysis identifying the significant downregulation of DNMT3A upon loss of MyoD. **(G)** Expression levels of DNMT1, DNMT3A, and DNMT3B in RH5 MyoD$^{\Delta}$ compared to RH5 Vector control cells, n = 3. **(H)** Biological replicates of ATAC-seq data of DNMT3A. Differences in the chromatin accessibility signal is highlighted in RH30 MyoD$^{\Delta}$ (purple) compared to RH30 Vector cells (blue), n = 3. Signal is represented as reads per million mapped reads. Data with error bars are depicted as mean ± SEM, * $p < 0.05$; ** $p < 0.01$.

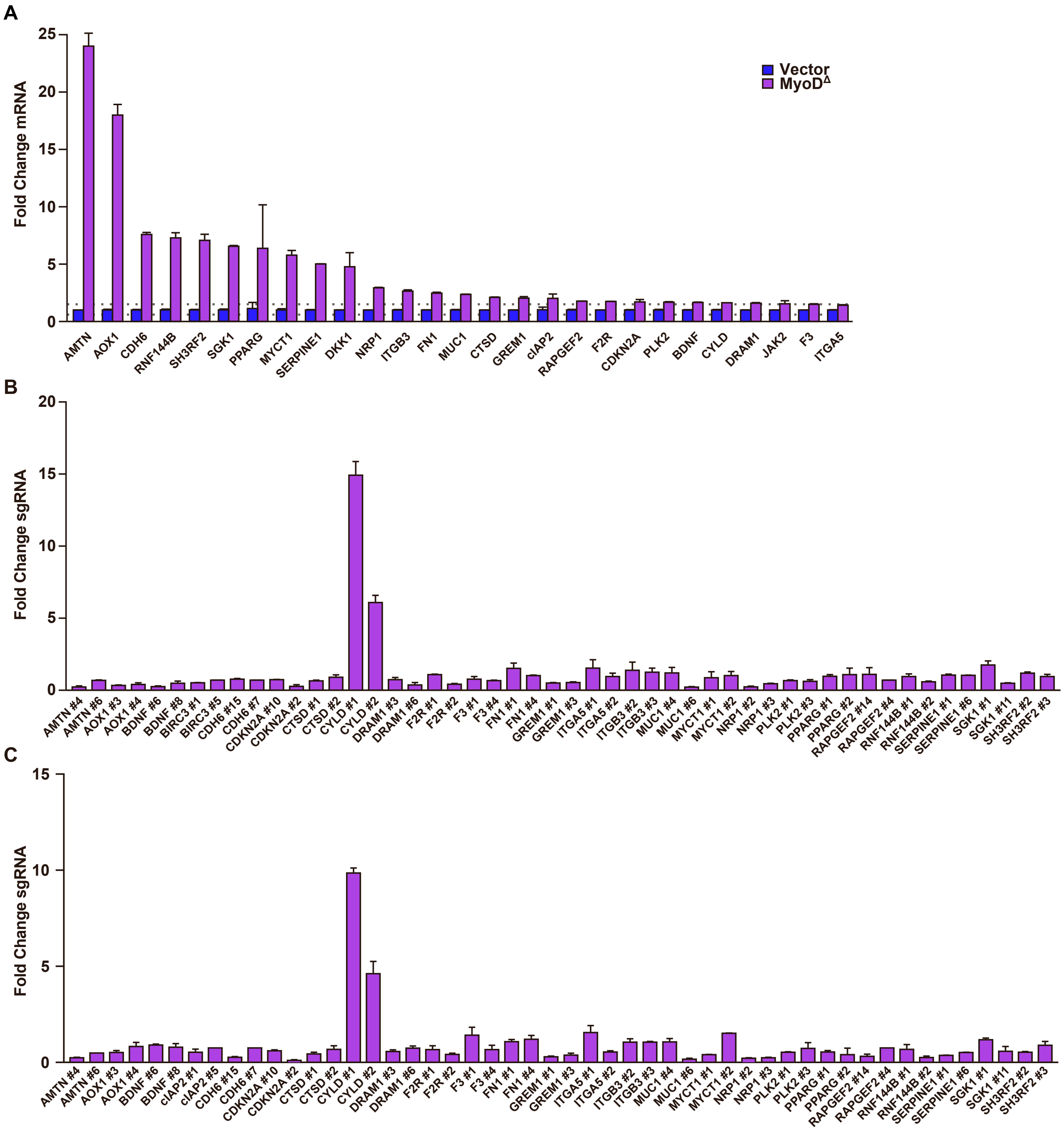
A
Fold Change mRNA
25
20
15
10
5
0
Vector
MyoD$^{\Delta}$
AMTN
AOX1
CDH6
RNF144B
SH3RF2
SGK1
PPARG
MYCT1
SERPINE1
DKK1
NRP1
ITGB3
FN1
MUC1
CTSD
GREM1
cIAP2
RAPGEF2
F2R
CDKN2A
PLK2
BDNF
CYLD
DRAM1
JAK2
F3
ITGA5
B
Fold Change sgRNA
20
15
10
5
0
AMTN #4
AMTN #6
AOX1 #3
AOX1 #4
BDNF #6
BDNF #8
BIRC3 #1
BIRC3 #5
CDH6 #15
CDH6 #7
CDKN2A #10
CDKN2A #2
CTSD #1
CTSD #2
CYLD #1
CYLD #2
DRAM1 #3
DRAM1 #6
F2R #1
F2R #2
F3 #1
F3 #4
FN1 #1
FN1 #4
GREM1 #1
GREM1 #3
ITGA5 #1
ITGA5 #2
ITGB3 #2
ITGB3 #3
MUC1 #4
MUC1 #6
MYCT1 #1
MYCT1 #2
NRP1 #2
NRP1 #3
PLK2 #1
PLK2 #3
PPARG #1
PPARG #2
RAPGEF2 #14
RAPGEF2 #4
RNF144B #1
RNF144B #2
SERPINE1 #1
SERPINE1 #6
SGK1 #1
SGK1 #11
SH3RF2 #2
SH3RF2 #3
C
Fold Change sgRNA
15
10
5
0
AMTN #4
AMTN #6
AOX1 #3
AOX1 #4
BDNF #6
BDNF #8
cIAP2 #1
cIAP2 #5
CDH6 #15
CDH6 #7
CDKN2A #10
CDKN2A #2
CTSD #1
CTSD #2
CYLD #1
CYLD #2
DRAM1 #3
DRAM1 #6
F2R #1
F2R #2
F3 #1
F3 #4
FN1 #1
FN1 #4
GREM1 #1
GREM1 #3
ITGA5 #1
ITGA5 #2
ITGB3 #2
ITGB3 #3
MUC1 #4
MUC1 #6
MYCT1 #1
MYCT1 #2
NRP1 #2
NRP1 #3
PLK2 #1
PLK2 #3
PPARG #1
PPARG #2
RAPGEF2 #14
RAPGEF2 #4
RNF144B #1
RNF144B #2
SERPINE1 #1
SERPINE1 #6
SGK1 #1
SGK1 #11
SH3RF2 #2
SH3RF2 #3

D

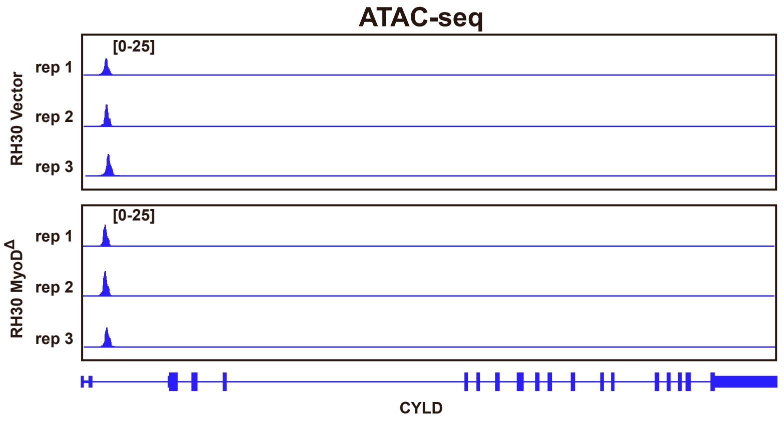

E

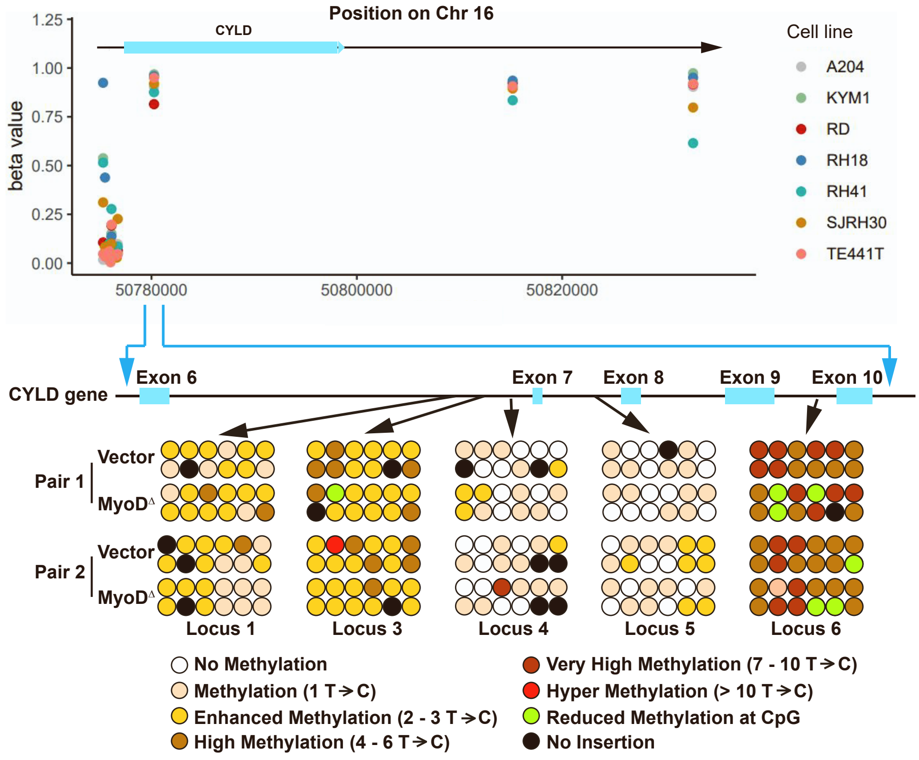

Figure S6. CYLD is epigenetically repressed by MyoD in RMS cells, related to Figure 6. (A) Magnified view from Figure 6A of qPCR analysis of 25/77 death related genes in RH30-SR MyoD$^{\Delta}$ cells compared to Vector control >1.5 mRNA fold change. **(B)** PCR results showing enrichment of targeting sgRNA guides in RH30-SR MyoD$^{\Delta}$ cells infected with 1 virus particle per cell following 4 cycles of TNF (10 ng/mL) treatment for 24 hrs compared to control untreated cells, represented as technical duplicates; n = 1. **(C)** PCR results showing enrichment of targeting sgRNA guides in RH30-SR MyoD$^{\Delta}$ cells infected with multiples virus particles per cell following 2 cycles of TNF (10 ng/mL) treatment for 48 hrs compared to control untreated cells, represented as technical duplicates; n = 1. **(D)** ATAC-seq of CYLD in RH30 MyoD$^{\Delta}$ compared to RH30 Vector cells, n = 3. **(E)** (Upper) Cancer Cell Line Encyclopedia (CCLE) data set investigation of methylation status and probe location on the CYLD gene. (Lower) Bisulfite sequencing was performed on RH30 MyoD$^{\Delta}$ compared to RH30 Vector cells, n = 3. Methylation status is depicted from different clones with varying methylation levels. Data with error bars are depicted as mean ± SEM.

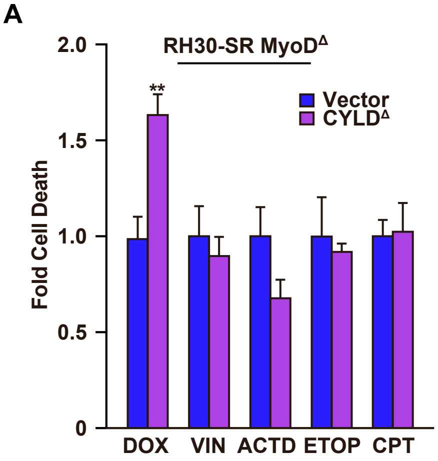

Figure S7. Deletion of CYLD is unable to rescue RMS cells lacking MyoD in the presence of chemotherapeutic stress, related to Figure 7. (A) RH30-SR MyoD$^{\Delta}$ Vector and RH30-SR MyoD$^{\Delta}$ CYLD$^{\Delta}$ cells were treated with DOX (1 µM), vincristine (VIN, 10 nM), actinomycin D (ACTD, 10 nM), ETOP (10 µM), or CPT (10 µM) for 24 hrs and subsequently analyzed for cell death; n = 4. Data with error bars are depicted as mean ± SEM, ** $p < 0.01$.

Table S1. Myogenic gene distribution, related to Figure S2A

Genes	Mean	Standard Error	95% Conficence interval lower	95% confidence interval upper
MyoD	1.083	0.046	0.992	1.175
Myogenin	1.215	0.087	1.042	1.388
Tnnt	1.083	0.046	0.991	1.174
Myosin Heavy Chain	1.088	0.047	0.994	1.183
GAPDH	1.109	0.066	0.978	1.24

Table S2. Positions of amplification loci on CYLD gene, related to Figure S6

No.	IlmnID	CHR	MAPINFO	Start of CYLD gene	Location on CYLD gene seq	PCR Locus	Location on gene
1	cg27588321	chr16	50775288	50694216	81072	1	Intron 6
2	cg20769774	chr16	50775476	50694216	81260	1	Intron 6
3	cg12691198	chr16	50775626	50694216	81410	2	Intron 6
4	cg04982619	chr16	50775666	50694216	81450	2	Intron 6
5	cg05340489	chr16	50775671	50694216	81455	2	Intron 6
6	cg00131870	chr16	50775820	50694216	81604	3	Intron 6
7	cg04986694	chr16	50775824	50694216	81608	3	Intron 6
8	cg15634032	chr16	50775878	50694216	81662	3	Intron 6
9	cg08935793	chr16	50776015	50694216	81799	4	Intron 6
10	cg01408432	chr16	50776024	50694216	81808	4	Intron 6
11	cg06458795	chr16	50776026	50694216	81810	4	Intron 6
12	cg02663864	chr16	50776102	50694216	81886	4	Intron 6
13	cg12637205	chr16	50776628	50694216	82412	5	Intron 7
14	cg08402842	chr16	50776714	50694216	82498	5	Intron 7
15	cg09722826	chr16	50780248	50694216	86032	6	Intron9
16	cg01646516	chr16	50815179	50694216	120963	7	>18kb behind CYLD related to H3K4me1 hESC enhancer
17	cg02451467	chr16	50832749	50694216	138533	8	>30kb behind CYLD gene.
